# A novel piperazine derivative that targets hepatitis B surface antigen effectively inhibits tenofovir resistant hepatitis B virus

**DOI:** 10.1038/s41598-021-91196-1

**Published:** 2021-06-03

**Authors:** S. Kiruthika, Ruchika Bhat, Rozaleen Dash, Anurag S. Rathore, Perumal Vivekanandan, B. Jayaram

**Affiliations:** 1 Kusuma School of Biological Sciences, Indian Institute of Technology, Delhi, New Delhi India; 2 Department of Chemistry, Indian Institute of Technology, Delhi, New Delhi India; 3 Supercomputing Facility for Bioinformatics and Computational Biology, Indian Institute of Technology, Delhi, New Delhi India; 4 Department of Chemical Engineering, Indian Institute of Technology, Delhi, New Delhi India

**Keywords:** Hepatitis B virus, Antiviral agents, High-throughput screening, Virtual drug screening, Target identification, Target validation, Small molecules

## Abstract

Chronic hepatitis B virus (HBV) infection is a global problem. The loss of hepatitis B surface antigen (HBsAg) in serum is a therapeutic end point. Prolonged therapy with nucleoside/nucleotide analogues targeting the HBV-polymerase may lead to resistance and rarely results in the loss of HBsAg. Therefore, inhibitors targeting HBsAg may have potential therapeutic applications. Here, we used computational virtual screening, docking, and molecular dynamics simulations to identify potential small molecule inhibitors against HBsAg. After screening a million molecules from ZINC database, we identified small molecules with potential anti-HBV activity. Subsequently, cytotoxicity profiles and anti-HBV activities of these small molecules were tested using a widely used cell culture model for HBV. We identified a small molecule (ZINC20451377) which binds to HBsAg with high affinity, with a KD of 65.3 nM, as determined by Surface Plasmon Resonance spectroscopy. Notably, the small molecule inhibited HBsAg production and hepatitis B virion secretion (10 μM) at low micromolar concentrations and was also efficacious against a HBV quadruple mutant (CYEI mutant) resistant to tenofovir. We conclude that this small molecule exhibits strong anti-HBV properties and merits further testing.

## Introduction

Hepatitis B virus (HBV) causes chronic infection and can increase the risk of liver cancer. Oral nucleoside analogues and interferon injections are used for treating chronic HBV infection. Only 30–40% of chronic HBV (CHB) patients respond to interferon treatment^1^. Approved drugs against HBV are inhibitors of reverse transcriptase activity of the HBV polymerase. Tenofovir and entecavir are recommended by the World Health Organization (WHO) for the treatment of CHB^2,3^. Tenofovir is generally believed to have a very high genetic barrier to resistance as the drug has been successfully used for a few years without any documented resistance. In 2019, Park et al. identified a CYEI quadruple mutation (rtS106C [C], rtH126Y [Y], rtD134E [E], and rtL269I [I]) that conferred resistance to tenofovir in two patients; no approved therapies were effective in patients with this mutant^4^. With increasing usage of tenofovir worldwide for the treatment of CHB, the resistance to tenofovir is expected to rise rapidly. Therefore, the urgent need for new therapies for the tenofovir-resistant HBV is increasingly recognized^3^. Development of new antiviral agents targeting HBV proteins other than the polymerase may help improve therapeutic options for chronic infection.

HBV is a small (3.2 kb) enveloped DNA virus belonging to the *Hepadnaviridae* family consisting of four partially overlapping ORFs namely P, S, C, and X that encode a total of seven proteins. The surface (S) ORF encodes three surface proteins of different lengths using three in-frame initiation codons. All three HBV surface antigens (large, medium, and small) share a common S domain towards the C-terminal and are embedded in the viral envelope^5^. Hepatitis B surface antigen (HBsAg) is a multi-transmembrane protein found in N-glycosylated (asparagine-146 of the common S domain) and un-glycosylated forms which form homo- or hetero-dimer through disulfide linkage^6,7^. The small surface antigen is abundantly produced, and the excess protein produced can undergo multimerization to form non-infectious subviral particles (SVP) without a nucleocapsid^8^. SVPs are secreted in 10^3^–10^6^ fold excess compared to the infectious virion^9^. These SVPs can modulate host immune response.

In this study, we used computational methods to screen a million molecules from ZINC database that can target HBsAg. We identified five potential small molecule inhibitors against HBsAg in the initial computational screening. One of these small molecules (ZINC20451377) (Figure S1) binds HBsAg in vitro and reduced HBsAg levels and hepatitis B virion secretion in a widely used cell culture model for HBV. Furthermore, the small molecule inhibitor could efficiently inhibit two drug–resistant HBV-polymerase mutants including the CYEI mutant resistant to tenofovir and rtM204I mutant resistant to lamivudine. In summary, we have identified and validated a small molecule inhibitor that targets HBsAg resulting in the inhibition of hepatitis B virion secretion from wild-type and drug resistant HBV mutants.

## Results

### Sequence alignment and homology modelling

**Figure 1:**
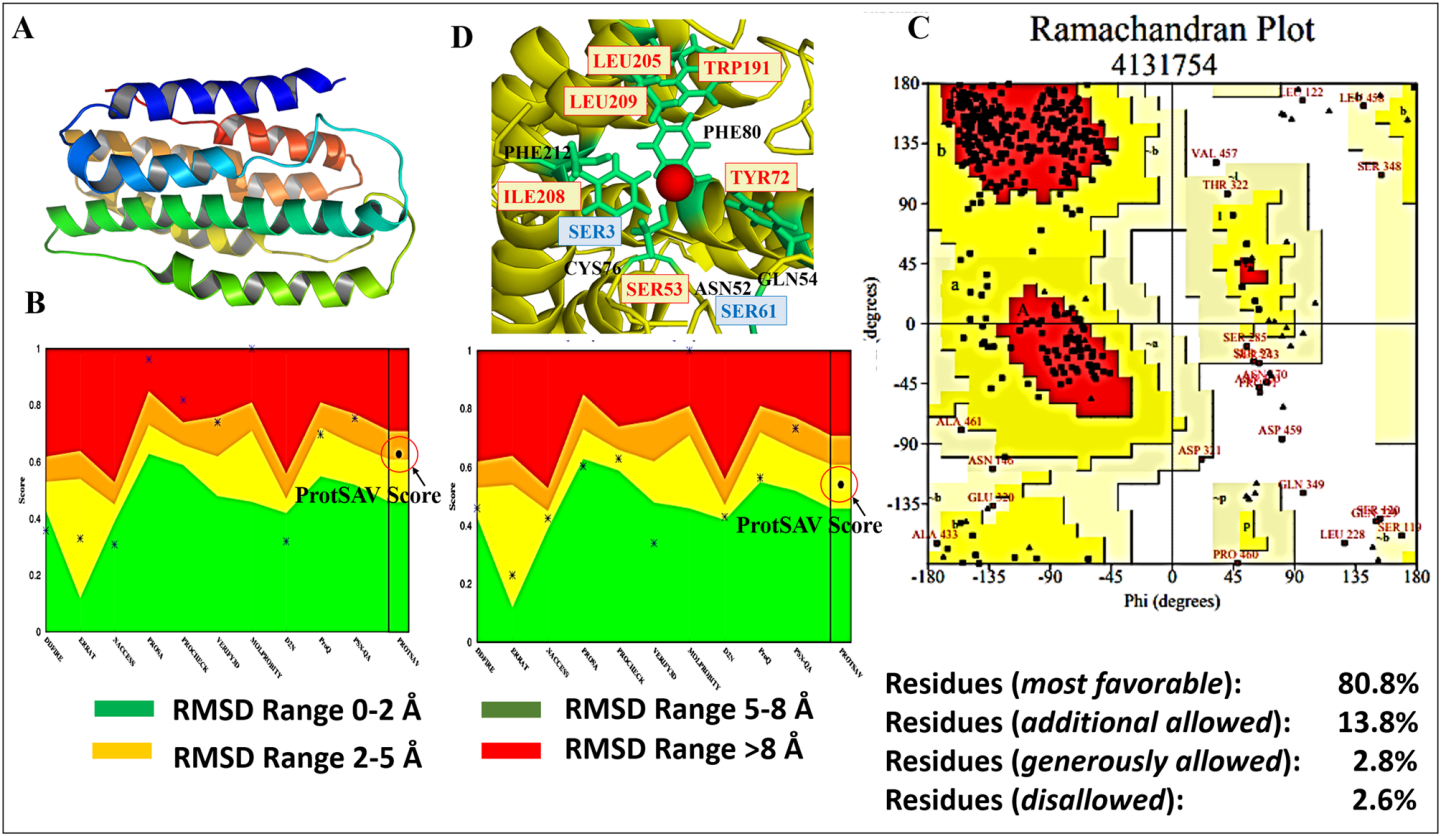
(**A**) The final selected structure of HBsAg obtained after structural refinement. (**B**) ProTSAV scores found earlier in the range from 5 to 8 Å and 2–5 Å after iterative refinement steps. (**C**) Ramachandran Plot for the selected final structure of HBsAg. (**D**) Active Site predicted for HBsAg via AADS showing its nearby residues.

The whole genome sequence of HBV genome was accessed from NCBI^10^ (NC_003977.2, strain *ayw*). No structure homology was identified for HBsAg while using BLASTP program against the PDB database. Tertiary structure modelling tools, BhageerathH+^11–13^ and I-TASSER^14^, were used to model the structure of HBsAg (Fig. 1A). These modelled structures were further optimized to the best energetically favourable conformation. Further, the modelled structures were refined using GalaxyRefine. The refined structures were subjected to molecular dynamics simulations consisting of minimization then heating followed by production of 20 ns. Snapshots of each nanosecond were selected and iteratively assessed computationally for their structural validation. The energetically favourable structures generated from these different simulations were assessed using ProTSAV (Fig. 1B). The structure which showed best ProTSAV score was further checked using RAMPAGE^15^ The Ramachandran plot for the final model was calculated which showed that only 2.8% of φ, ψ angles were in disallowed regions, 80.8% of the residues were in the most favoured region, and the remaining residues were in the additionally allowed regions (Fig. 1C). This ensured that the final structure can be used for structure-based drug design (Fig. 1B,C). It is interesting to note that our final modeled structure of HBsAg is analogous to the reported topological structures by others^7,16,17^. These studies suggest that HBsAg has a structure of membrane spanning helices, followed by loops in extracellular regions. This is seen in our final model as well (Figure S2). Other studies claiming that HBsAg has major percentage contribution (~ 45–50%) of helices further increases our confidence in the predicted structure^18–20^. The transmembrane domains (TM1, TM2, TM3 and TM4) and the cytoplasmic and luminal sides of the HBsAg protein are highlighted in the Figure S2 for better understanding of the HBsAg 3D model.

### Active site identification

The final structure selected was submitted to AADS program^21^ to predict its potential ten binding sites. The binding pocket that was ranked first by the AADS program was selected for screening and docking studies (Fig. 1D).

### Screening and docking studies

The ZINC database^22^ consisting of a million molecules was screened against the identified site in HBsAg. The top 150 ZINC molecules ranked based on their predicted binding affinities (Table S1) were further analysed using atomic level docking and scoring. Out of these top 150 ZINC molecules, 30 small molecules were selected based on their better binding energies obtained using ParDOCK, SWISS-Dock and AutoDock (Table S2). Further short-listing of five molecules was done based on high binding energies obtained using ParDOCK (Table S2). These five molecules also showed close interaction networks within the HBsAg cavity as shown in Table S3 and thus were subjected to short 10 ns MD simulations. However, molecules 1, 2 and 4 showed increase in RMSD after initial 8 ns whereas, molecules 3 and 5 showed stable trajectories till 10 ns. Thus, the comparative analysis of these five docked complexes based on their initial 10 ns MD simulations (Figure S3) yielded two small molecules (molecules 3 and 5) that were potential inhibitors of HBsAg.

### Cytotoxicity testing

Cell viability in the presence of increasing concentrations of molecules 3 and 5 was determined using MTT assay (Figure S4). The concentration of a given molecule at which at least 90% of cells were viable was selected for anti-HBV studies.

### Identification of molecule 5 for further testing

We analysed HBsAg levels in supernatant of Huh7 cells transfected with the 1.3 × HBV Wild type plasmid. Molecule 5 (ZINC20451377), but not molecule 3, inhibited HBsAg below the detection limit (Figure S5). Thus, only molecule 5 was selected for further analysis of anti-HBV activity. Cell viability in presence of increasing concentrations of molecule 5 was further assessed by additional assays including cell counting kit-8 (CCK-8) and resazurin reduction assays (Figure S6).

To ascertain that this molecule does not inhibit other proteins involved in normal cellular transcription or translation machinery, we transfected Huh7 constructs with a firefly luciferase reporter plasmid^23^ and renilla luciferase expression plasmid (control), then added 10 µM of molecule 5. The relative luciferase expression was comparable in cells with or without molecule 5 (Figure S7), thus ruling out inhibition of proteins involved in cellular transcription and translation machinery by this molecule.

### Characterization of binding kinetics of molecule 5 with HBsAg

**Figure 2:**
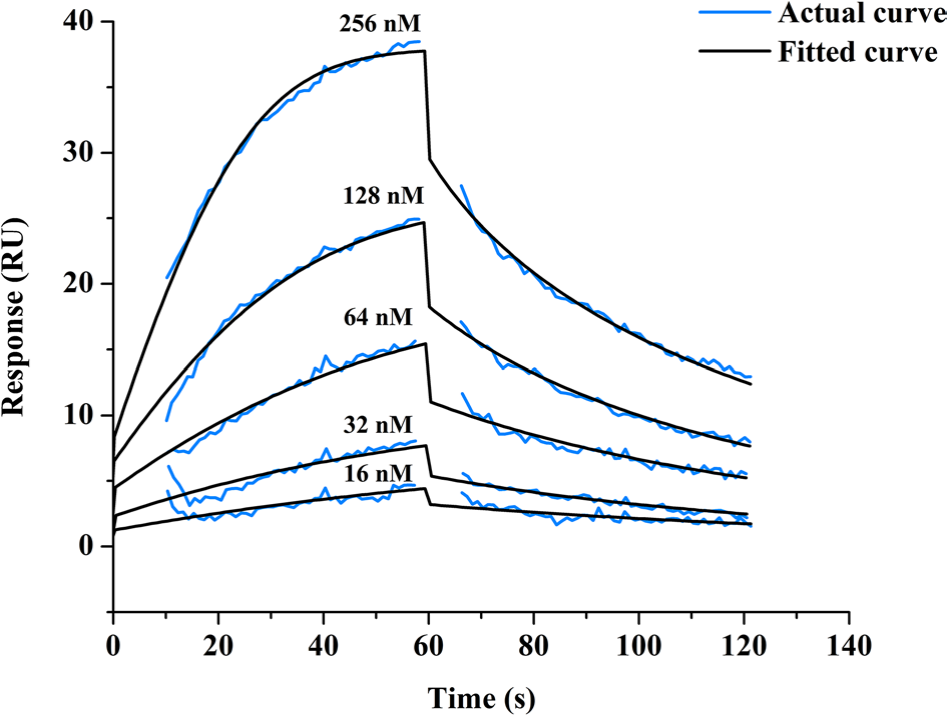
Surface plasmon resonance (SPR) analysis showing binding kinetics of Hepatitis B Surface Antigen (HBsAg) with molecule 5. Kinetic analysis of HBsAg-Molecule 5 binding was performed by injecting different known concentrations of molecule 5 (from 16 to 256 nM) over HBsAg immobilized carboxymethyl dextran-coated CM5 sensor chip (Amine-coupling chemistry). All measurements were performed at 25 °C with a flow rate of 30 μL/min using HBS-EP buffer with association time 60 s followed by 60 s dissociation phase. Kinetic constants were calculated from the sensorgrams using the 1:1 fit model using BIA Evaluation 2.0.1 (Cytiva) software. The blue line indicates the actual curve and black line indicates the fitted curve of the sensorgram.

To experimentally analyze the binding kinetics of computationally predicted molecule 5 with HBsAg, we performed surface plasmon resonance (SPR) spectroscopy. The equilibrium constant (KD) is the ratio of the dissociation rate (k_off_; how quickly it dissociates from HBsAg) to the association rate (k_on_; how quickly it binds to HBsAg). The KD value obtained from the SPR analysis is 6.53 × 10^–8^ M with the association rate constant of 6.21 × 10^5^ M^−1^ s^−1^ and dissociation rate constant of 0.04057 s^−1^. The smaller the KD value, the greater the binding affinity of the ligand for its target and vice-versa^24^. The binding affinities of varying concentrations of molecule 5 to HBsAg are shown in Fig. 2. For the negative control, we performed the binding kinetics study of ciclopirox with HBsAg. Ciclopirox is a synthetic antifungal drug which binds to the HBV core protein and not the HBV surface antigen^25^. In the control SPR experiment, no binding or negative binding affinity was observed (Figure S8). The statistical parameters Chi^2^ and U-value indicate the quality of fit. The parameters for goodness of fit and kinetic constants are reported in Table S4. A low U-value less than 15 (U-value 3, in case of Molecule 5) indicates greater confidence in the results^26,27^. Therefore, the KD value obtained for ciclopirox is erroneous (U-value 95) and indicates no binding with HBsAg.

### Molecule 5 inhibits HBsAg in a dose-dependent manner

**Figure 3:**
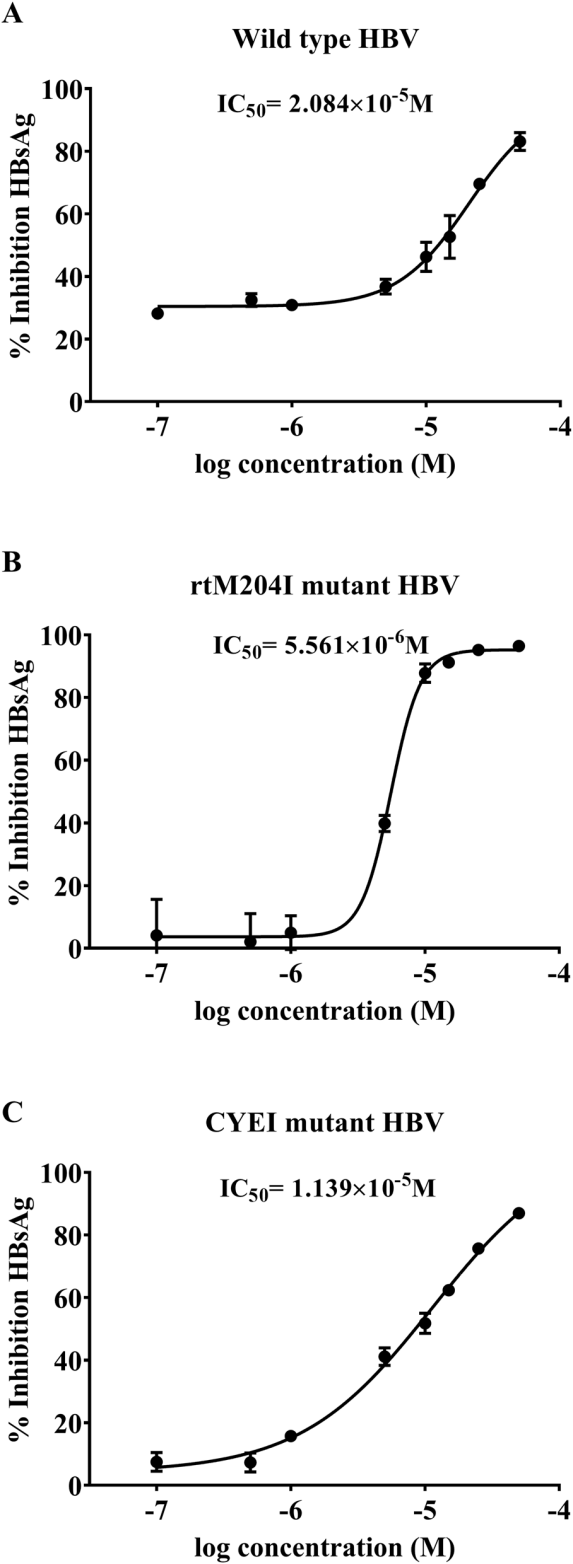
Dose dependent inhibition of secreted HBV surface antigen (HBsAg) by Molecule 5 encoded by (**A**) Wild type HBV and (**B**) lamivudine-resistant rtM204I mutant HBV and (**C**) tenofovir-resistant CYEI mutant HBV.

Molecule 5 inhibits HBsAg in a dose-dependent manner (Fig. 3). Interestingly, the efficacy of molecule 5 was comparable for the wild-type, lamivudine-resistant (rtM204I) mutant HBV and tenofovir-resistant (CYEI) mutant constructs. A control experiment with lamivudine was performed. As Lamivudine is a nucleoside analogue (NA) that targets the HBV polymerase, it is not expected to inhibit HBsAg levels. As expected, secreted HBsAg levels were unaffected by lamivudine (Figure S9). The IC_50_ values of molecule 5 for HBsAg are listed in Table S5. Computational ADMET properties are listed in Table S6.

As molecule 5 led to a dose-dependent decrease in the levels of secreted HBsAg, it is important to understand whether it is inhibiting the production of HBsAg or is it merely inhibiting the secretion of HBsAg. For this purpose, the intracellular HBsAg was quantified in the presence of molecule 5 (Figure S10A). Molecule 5 led to a decrease in the intracellular levels of HBsAg, which indicates it is not merely inhibiting the secretion of HBsAg.

### Inhibition of HBsAg by molecule 5 is independent of other HBV-encoded proteins

HBV encoded proteins may regulate each other or HBV replication^28–31^. We therefore wanted to test if the inhibition of HBsAg is independent of inhibition of other HBV encoded proteins. For this purpose, we transfected Huh7 cells with the sub-genomic surface antigen expression construct (PreS2/S region; please see methods section for details). Subsequently, the Huh7 cells were treated with molecule 5 for 48 h. Analysis of HBsAg levels in the supernatant and cell lysate using ELISA indicates that molecule 5 is able to inhibit HBsAg expression from sub-genomic expression construct (Figure S10B,C). This finding suggests that molecule 5-mediated downregulation of HBsAg is independent of other HBV encoded proteins.

### MD simulations of molecule 5 in complex with HBsAg

**Figure 4:**
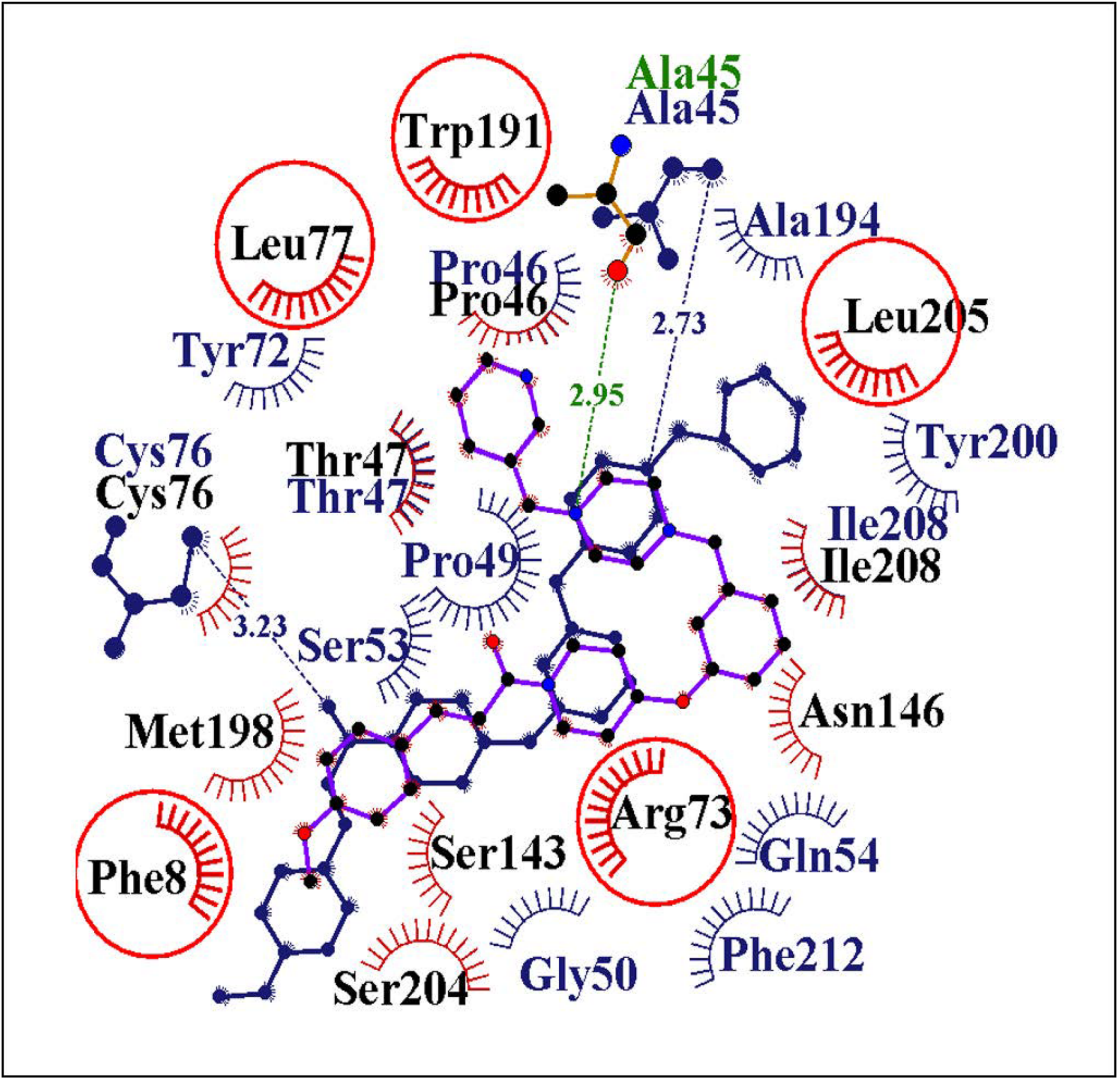
2D interaction patterns of molecule 5 are shown overlapped between the initial docked pose and the most favourable conformation (biggest cluster pose) during the 100 ns molecular simulations. Blue coloured residues indicate initial docked poses and the red coloured residues belong to biggest cluster. Green coloured residues are hydrogen bonding residues. The 2D diagram shows that the van der Waals interactions are very strong as the common residues at the initial and biggest cluster pose are maximum in number.

The best pose conformations between molecule 5 and HBsAg were visually ascertained and subjected to MD simulations for 100 ns. The molecule showed promising results in terms of the computationally predicted binding energies as well (Table S7). The hydrogen bonding and hydrophobic contact patterns within the predicted active site of HBsAg are shown in Fig. 4. Ligand RMSD and protein backbone analysis showed overall stability. The regions of most favourable conformation of molecule within the active site of HBsAg during the 100 ns simulations (Figure S11) as seen from the RMSD plots were clustered. The initial docking pose and the biggest cluster pose found from one of the frames where the convergence plots showed stable trajectory, were overlapped (Fig. 4). The most favourable poses during the 100 ns trajectory analyses showed that most of the amino acid residues such as Cys76, Trp191, Leu77, Leu205, Arg73, Ile208, Pro46, Thr47 maintained their hydrophobic contacts with the molecule 5 during the simulation runs of 100 ns. However, residue Ala45 exhibited a constant hydrogen bonding with molecule 5 throughout the 100 ns simulations.

### Binding mode analysis of molecule 5

**Figure 5:**
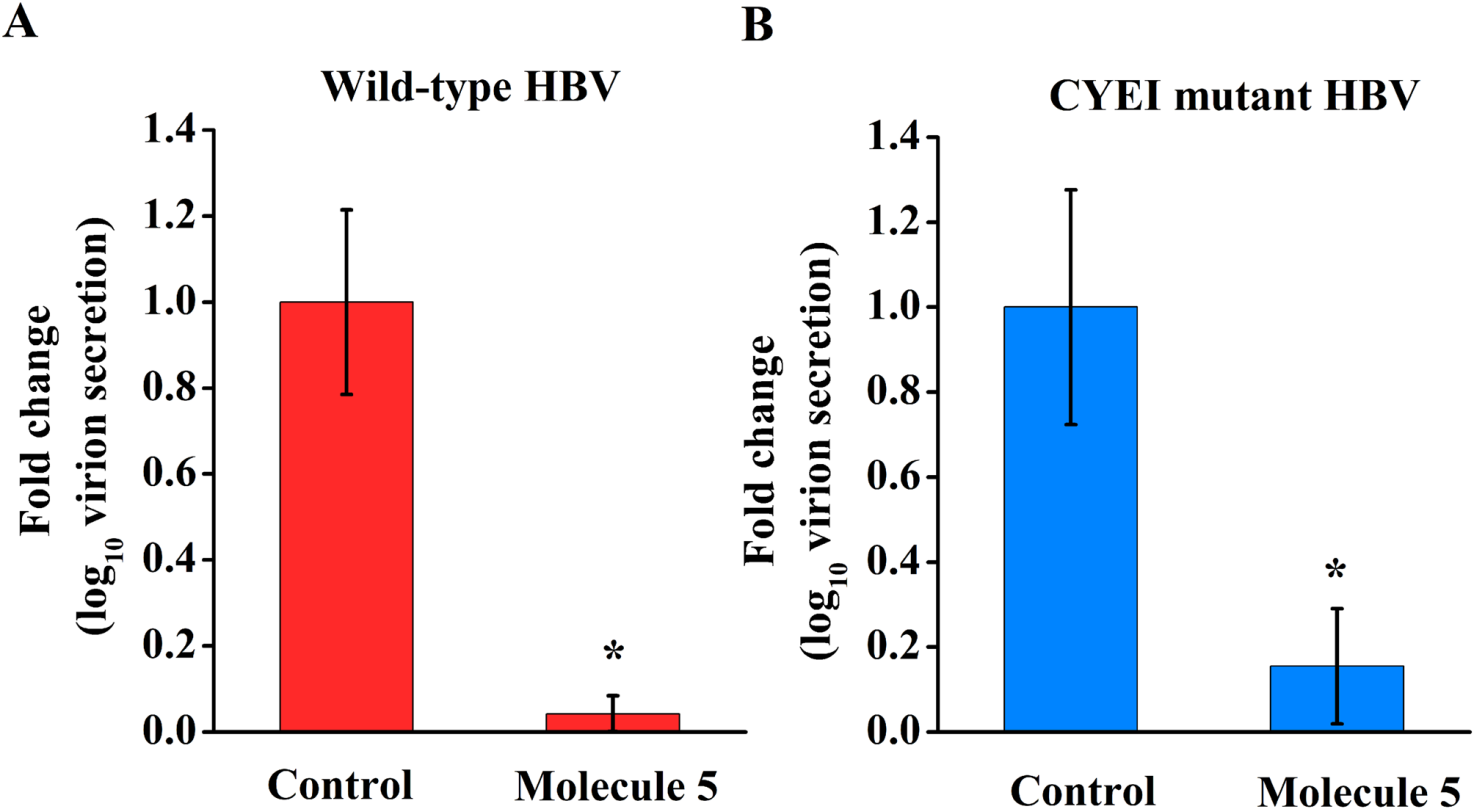
Inhibition of Secreted hepatitis B virion (log_10_ copies)/ml supernatant (**A**) Wild-type HBV and (**B**) Tenofovir resistant CYEI mutant HBV by 10 µM Molecule 5 normalised to control was estimated using real-time PCR following virion capture as described in the methods section. Values significantly different from controls are indicated by a Student’s t-test where, *p < 0.05.

To better understand the mechanism of action of the best binding molecule 5, we carried out an analysis of the complexes over the 100 ns simulations within the binding pocket of HBsAg. For molecule 5, the interaction patterns show that the hydrogen bonds stay stable throughout the MD simulations along with a tight network of van der Waals interactions with proximal residues (Figs. 4, S11 and S12). Molecule 5 bound to HBsAg complex, the Cys76 association with molecule 5 turns out to be weak during the 100 ns molecular dynamics simulations and only backbone O atom of Ala45 interacts with molecule 5 throughout the 100 ns run (Figs. 4 and S12). However, the overall stability of the molecule 5 within the pocket of HBsAg is observed with a tight network of van der Waals interactions with residues Pro46, Thr47, Arg73, Cys76, Leu77, Trp191, Leu205 and Ile208 throughout the 100 ns simulations (Fig. 5). Thus, molecule 5 acts as a good binder for HBsAg.

### Molecule 5 inhibits hepatitis B virion secretion

Molecule 5 was associated with significantly reduced hepatitis B virion secretion in cell culture (Fig. 5). Molecule 5 led to inhibition of hepatitis B virion secreted from the wild-type HBV and CYEI mutant constructs. Lamivudine was used as a positive control for virion secretion experiments (Figure S13). Virion secretion for the rtM204I mutant could not be assessed as the levels were below the limit of detection of the virion secretion assay^32^. Previous reports indicate that the rtM204I mutant may be associated with impaired virion secretion^33,34^. Virion secretion from a stable HBV expressing cell line HepG2.2.15 was inhibited after molecule 5 treatment (Figure S14). The reduction in virion secretion is more pronounced than the reduction in HBsAg levels. This is in keeping with previous reports where a minor reduction in key components of the virion such as HBsAg has been associated with more pronounced reduction in virion secretion^23^.

### HBV covalently closed circular DNA (cccDNA) levels remain unchanged after molecule 5 treatment

In this study, we have used plasmids expressing overlength copies of HBV (1.3 × HBV and 1.2 × HBV) and cccDNA cannot be determined efficiently in overlength plasmids because of background signal from the transfected plasmid (i.e., all transfected DNA is completely double stranded and since it is more than unit length, the transfected DNA will be positive in HBV cccDNA assays). Hence, HepG2.2.15 cell line which forms cccDNA^35–37^ was used to assess the effect of molecule 5 on cccDNA levels. HBV cccDNA levels remained unaltered after molecule 5 treatment (Figure S15) which indicates a direct effect of molecule 5 at the protein level^38^.

### Comparison of molecule 5 with other known inhibitors

Benzimidazole, nicotinamide, biphenylamide, cyclophilin A, phenylpropenamide, mycophenolic acid and nucleoside analogues have been reported as HBV inhibitors^39,40^. In contrast, molecule 5 discussed in the present study is a piperazine derivative. The piperazine moiety of molecule 5 is predicted to be involved in hydrogen bonding and in van der Waals interactions with the target (HBsAg) in our 100 ns molecular simulations and hydrogen bond analysis (Fig. 5). Therefore, we believe that in addition to previously reported inhibitors of HBV, piperazine moiety may offer a new scaffold for HBV inhibition. To analyse the novelty of the scaffolds identified in our study, the Tanimoto coefficient of similarity was calculated between the small molecule 5 and the previously reported secretion inhibitors of HBsAg such as HBF-0259, BM601, Nicotinamide and NJK14047^39,40^ (Table S7). The coefficient values showed very low levels of similarity, thus establishing that in this study we have identified a novel scaffold against HBsAg. Of note, all previously reported HBsAg inhibitors are secretion inhibitors and inhibition of HBsAg secretion may lead to increased accumulation of HBsAg within infected cells^39,40^. Immune response to HBV proteins are the major cause of liver pathology in chronic HBV infection^41,42^. Therefore, molecule 5 not only represents a novel scaffold but also represents the yet unknown class of anti-HBV molecules that inhibit intracellular and extracellular (secreted) HBsAg levels.

## Discussion

**Figure 6:**
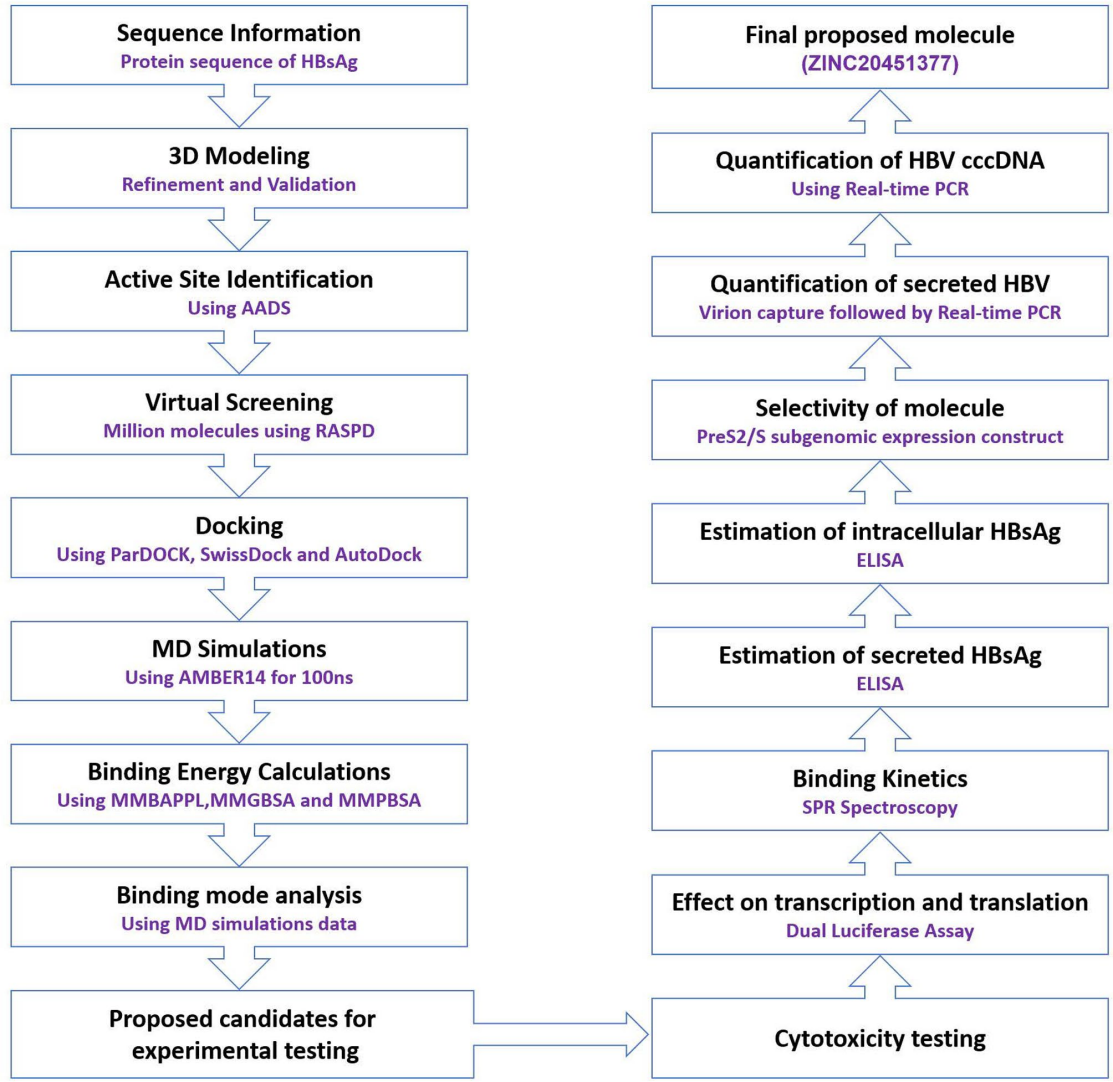
Flowchart of the workflow used for identification of molecule 5 (ZINC20451377) as an inhibitor of HBsAg that results in inhibition of hepatitis B virions.

In this study, we used computational methods and identified commercially available small molecules targeting HBsAg. In the absence of an experimental structure for HBsAg, we used hybrid modelling tools to predict the 3D framework of this protein. With further refinement of the predicted model using molecular dynamics simulations^43–47^, we were able to find a tertiary structure having the structural parameters within acceptable ranges. After exhaustive computational studies we identified a few molecules that exhibit inhibitory potential against HBsAg. Among them, molecule 5 was identified to independently inhibit HBsAg (Figures S10B,C) without interfering with the normal cellular transcription or translation machinery, in the HBV cell culture model (Figure S7). The workflow used for this study has been shown in Fig. 6.

The goals of treatment for chronic Hepatitis B (CHB) are the loss of secreted HBsAg and undetectable viral load. Treatment with nucleoside analogues can lead to loss of HBV DNA, but rarely leads to the loss of HBsAg, a desired treatment endpoint. Therefore, CHB patients have to undergo long-term antiviral treatment. But, the emergence of drug resistance while treating with nucleoside analogues appears to be inevitable. Among the anti-HBV drugs that are currently used, tenofovir has been used for long durations without the emergence of drug resistance. However, recent identification of tenofovir-resistant mutants in patients highlights the urgent need for new therapies for CHB. Currently, no FDA approved drugs have been successful used in clinical settings to inhibit the tenofovir-resistant CYEI mutant. Using a cell culture model, Park et al*.* (2019) reported that the CYEI mutant was susceptible to a capsid assembly modulator*,* which can act as rescue therapy for patients with tenofovir-resistant HBV^4^. Development of new antiviral strategies targeting HBV proteins other than the HBV polymerase is particularly important as there are no FDA approved drugs in this category.

HBsAg forms dimers shortly after it is produced in the endoplasmic reticulum (ER). HBsAg is co-translationally inserted into the ER and glycosylated in Golgi complex^7^. This process is controlled by the ER quality control system and 10% of HBsAg produced remains inside the hepatocyte which undergoes ER-associated degradation (ERAD)^48,49^. HBsAg stays in the ER for hours^7^, which provides the small molecule extended amount of time to act on the target protein^49^.

Nucleozin, a small molecule that inhibits influenza virus replication inhibits virus RNA synthesis, virus protein synthesis and oligomerization of the nucleoprotein^50^. This is an example of a small molecule inhibitor that targets several aspects of virus replication. Similarly, several markers of HBV replication are inhibited by small molecule inhibitors^51^. These findings demonstrate how small molecules targeting a single viral protein may inhibit multiple aspects of virus replication. Mechanisms of small molecule-mediated inhibition of virus replication may not be fully understood despite their efficacy in vitro and in vivo^52^. In this work, we experimentally demonstrate that Molecule 5 binds HBsAg leading to a dose-dependent reduction in HBsAg levels and inhibition of hepatitis B virion production in cell culture. However, we have not elucidated the specific underlying mechanisms. Small molecule inhibitors have been shown to facilitate degradation of virus proteins^49,53^, inhibit envelope protein maturation and incorporation into virus particles^49^, inhibit virus RNA synthesis^52^, alter intracellular localization of virus proteins or inhibit interaction among essential virus proteins^54^.

In summary, we have identified a novel small molecule which leads to inhibition of intracellular HBsAg resulting in reduced secretion of HBsAg and hepatitis B virion (10 μM) at low micromolar concentrations. Furthermore, the efficacy of molecule 5 is comparable for wild-type HBV, a lamivudine-resistant mutant (rtM204I) and a tenofovir-resistant mutant (CYEI) HBV. We conclude that molecule 5 can inhibit wild-type and drug resistant HBV and merits further testing.

## Materials and methods

### Protein target identification and structural analysis

The full-length HBV genome of subtype *ayw* NC_003977.2 from NCBI database was used for analysis. The protein sequence (YP_009173871.1) of HBsAg (226 amino acids long) was imported into BLASTP program^55^ against PDB to find its homolog template structures. The three-dimensional structure of HBsAg was generated using BhageerathH+^11,13^ and I-TASSER^14^. The modelled structures were optimized using the structural refinement tool Galaxy Refine^45^. Further optimization of the structures was carried out by MD simulations with AMBER14^56–58^. The modelled structures were subjected to minimization and production runs, for 20 ns and the snapshots were obtained from the most energetically favourable poses during the simulations with manual iterative structural validations. The structures having energetically favourable conformations among all the simulations were validated using ProTSAV^59^ and Ramachandran Maps^60^.

### Active site identification, screening, and docking studies

Active sites were identified using the active site prediction tool AADS^21^. Virtual screening of a million-molecule library^22^ was done using a rapid screening methodology, RASPD^61^ and the top 150 hits were further evaluated using the docking program ParDOCK^62^, SwissDock^63^ and AutoDock^64^. The binding energy calculated via ParDOCK (kcal/mol) was considered for narrowing down the search space for lead molecules. The best binding poses of lead molecules in complex with HBsAg were subjected to first short molecular dynamics simulations of 10 ns following further extension of molecular dynamics simulations till 100 ns for the better binding complexes.

### Molecular dynamics simulations

All of the simulation related calculations were performed using AMBER14^56–58^. The ff14SB AMBER force field^65^ was utilized for assigning partial atomic charges, van der Waals and bonded parameters to the protein atoms of the best binding docked complex. For creating the parameters and charge library (AMBER *prepin* and *frcmod* files) of the inhibiting molecules, the antechamber module of AMBER was used with GAFF^66^ and AM1BCC^67^, respectively. To understand the mechanism of binding and interactions between the inhibitor and the protein, the docked complex was provided with an environment of adequate number of ions and water molecules. The complex was first neutralized by addition of required numbers of Na^+^ and Cl^−^ ions to account for the flexibility of the ligand and the active site residues followed by solvation with an octahedral box of 12 Å thick TIP3P model layer of water around the complex. The initial topology and input coordinate files having information of ligands within the protein active site as in their best identified docked poses, along with water and ions were thus generated using Xleap module of AMBER^56^. The prepared system was then subjected to minimization of solvent molecules alone for 5000 steepest descent and 5000 conjugate gradient steps. This was followed by minimization of the whole solute–solvent system for 3000 steepest descent and 3000 conjugate gradient steps. This was performed to re-orientate water molecules into a lower energy geometry and bring the whole system to a stable energy minimized state. The energy minimized system was then heated gradually in NVT ensemble up to 300 K while subjecting the protein-inhibitor complex to harmonic restraints of 25 kcal/mol. This was done to bring the whole system gradually to the desired temperature of 300 K. Afterwards, the system was equilibrated in NPT ensemble at 300 K and a pressure of 1 bar using the Berendsen barostat while releasing the harmonic restraints on the complex from 5 to 0.1 kcal/mol, in six steps of 50 ps each. These equilibration steps allowed the water to equilibrate around the solute and come to an equilibrium density. Finally, all restraints were released and 10 ns production run in NPT ensemble (P = 1 bar, T = 300 K) was carried out in explicit solvent with periodic boundary conditions. A non-bonded cut-off of 8 Å was used in all the above calculations. The complexes with less fluctuating RMSD with reference to their respective minimized docked positions were extended for 100 ns production run in NPT ensemble. The overall binding free energy of the protein-inhibitor complexes throughout the trajectories was calculated using MMBAPPL^68^, MMGBSA and MMPBSA^69,70^. All the simulated complexes were monitored for their RMSD, energy (kinetic, potential and total), density and temperature fluctuations throughout the simulations to ensure that the standard deviations of each of these values were within acceptable limits.

### Binding free energy calculations

The overall binding free energies of the protein-inhibitor complexes throughout the trajectories were calculated using MMBAPPL^68^, MMGBSA, and MMPBSA^70,71^. MMGBSA and MMPBSA are popular approaches where the molecular mechanics energies are combined with the Poisson–Boltzmann or generalized Born and surface area continuum solvation models to get an estimate of binding free energies of small ligands to biological macromolecules. MMBAPPL is an empirical scoring function to predict the binding free energies of protein ligand complexes. These scores/ predicted binding energies when averaged over each snapshot from molecular dynamics simulations, provide more reliable semi-quantitative understanding of the inhibitory potential of a molecule.

### Molecules

Molecules 3 and 5, identified from the ZINC database, were procured from Chembridge (http://www.chembridge.com). Ciclopirox and lamivudine were purchased from Sigma Aldrich.

### Cytotoxicity testing

Huh7 cells were seeded overnight in a standard 96-well plate at a density of 5000 cells per well. Fresh media containing increasing concentrations of molecules dissolved in cell culture grade DMSO (0.5% final concentration) was added to cells and incubated for 48 h. MTT assay was performed as described previously^72^. Cell counting kit-8 (CCK-8) and resazurin reduction assays were performed as per the manufacturer’s (Sigma-Aldrich) suggested protocol.

### Plasmid constructs

HBV preS2/S region was cloned into pGEMT easy vector (Promega) to create a sub-genomic surface construct expressing the middle (M) and small (S) surface antigen. This construct was used to assess the ability of small molecule to inhibit HBsAg (or small surface antigen).

CMV promoter was cloned into a promoter less firefly luciferase vector (pGL3 basic vector) and a renilla luciferase reporter construct with a thymidine kinase promoter (pRL-TK, Promega) was used for the dual luciferase assays to rule out generic inhibition of the cellular transcription or translation apparatus by small molecule 5.

The wild-type and lamivudine resistant mutant (rtM204I) 1.3 × HBV plasmids (genotype D) were kindly provided by Dr. Syed Naqui Kazim (Jamia Millia Islamia, India)^73^. The tenofovir resistant CYEI quadruple mutant (genotype C)^4^ was a kind gift from Prof. Kyun-Hwan (Konkuk University, South Korea).

### Dual luciferase assay

Huh7 cells were seeded overnight in a standard 96-well plate (BD Falcon) at a density of 2 × 10^4^ cells per well. A reporter plasmid (CMV-Firefly Luciferase construct) and a control plasmid (TK-Renilla luciferase) were transfected in the ratio of 10:1, using Lipofectamine 2000. Six hours post-transfection, media containing 10 μM molecule 5 dissolved in DMSO (0.5% final concentration) were added to the cells, and Dual-luciferase assay (Promega) was performed using a standard protocol^74^. The luminescence from Firefly and Renilla luciferase were measured using a microplate counter (Microbeta2 microplate counter, PerkinElmer).

### Surface plasmon resonance (SPR) spectroscopy

Purified hepatitis B surface antigen (Cat No. FHBd0346, generously gifted to us by Mr. Laxminarayan, Yashraj Biotechnology Ltd, Mumbai, India) was used for surface plasmon resonance experiment^75^. The binding kinetics of HBsAg to molecule 5 or ciclopirox was determined by Biacore X100™ (Cytiva). Samples were dialyzed against running buffer (HBS-EP buffer, pH 7.4), HBsAg was immobilized on carboxy-methyl dextran-coated CM5 sensor chips (Cytiva) by Amine coupling method according to the manufacturer's recommendation^76^. Samples were injected in a series of concentrations ranging from 16 to 256 nM with association time 60 s followed by 60 s dissociation phase. For regeneration, 10 mM Glycine (pH 1.5) was used. All measurements were performed at 25 °C with a flow rate of 30 μL/min using HBS-EP buffer, as per the manufacturer’s protocol. Kinetic constants were calculated from the sensorgrams using the 1:1 fit model using BIA Evaluation 2.0.1 (Cytiva) software.

### Transfection of HBV constructs into Huh7 cells

Huh7 cells were seeded overnight at a density of 5 × 10^4^ cells per well in a 48-well plate and transfected with HBV construct using Lipofectamine 2000. Six hours post-transfection, media containing molecules dissolved in DMSO (0.5% final concentration) was added; culture supernatant and cells were harvested at 48 h. Transfections were performed in triplicates.

### Estimation of HBsAg

Secreted and intracellular HBsAg levels were estimated using MONOLISA HBsAg Ultra ELISA kit (BioRad) as described earlier^23,28^. Cells transfected with HBV construct and treated with DMSO (0.5% final concentration) were used as controls. Percentage Inhibition was calculated using absorbance value of DMSO as control. ELISA was performed in triplicates and data are represented as Mean ± SD and the plots were generated using GraphPad Prism software version 8.4.3. Nonlinear regression curve fitting was performed and IC_50_ values were calculated using log(inhibitor) vs response-variable slope (four parameters) equation.

### Transfection of PreS2/S subgenomic expression construct

Huh7 cells were transfected with a plasmid expressing only the PreS2/S region using Lipofectamine 2000 in a standard 24-well plate (BD Falcon). Six hours post-transfection, cells were treated with media containing 10 μM molecule 5 dissolved in DMSO (0.5% concentration). The culture supernatant was harvested 48 h post exposure. HBsAg levels were estimated as described earlier.

### Quantification of secreted HBV

Huh7 cells were seeded overnight at a density of 5 × 10^5^ cells per well and HepG2.2.15 cells were seeded at 8 × 10^5^ cells per well in a standard 6-well plate (BD Falcon). Huh7 cells were transfected with 1.3 × HBV wild type or 1.2 × HBV CYEI mutant construct using Lipofectamine 2000. Media containing 10 μM molecule 5 dissolved in DMSO (0.5% final concentration) were added to Huh7 cells (six hours post-transfection) and HepG2.2.15 cells (after overnight seeding). The culture supernatant was harvested after 72 h for quantification of secreted virion. Supernatants from the cells transfected with the HBV construct and treated with DMSO (vehicle control) were used as controls. Supernatants from Huh7 cells transfected with wild type 1.3 × HBV and treated with 5 µM lamivudine were used as positive control for inhibition of virion secretion^32^. Secreted virion in the culture supernatant were quantified using an assay developed in-house^32^. Briefly, the culture supernatants were added to commercially available HBsAg ELISA plate coated with polyclonal anti-HBsAg antibody (MONOLISA, BioRad) for immunocapture of the virus. The virion-associated DNA from the captured virion was extracted using the QIAamp DNA mini kit (Qiagen). Quantification of secreted virions was done by real-time PCR using FastStart essential DNA green master (Roche) and primers specifically designed to amplify the virion-associated DNA, FP: 5′-GGTCTGCGCACCAGCACC-3′ and RP: 5′-GAACTTTAGGCCCATATTAGTG-3′. A standard curve was generated for absolute quantification^32^.

### Quantification of HBV cccDNA

HepG2.2.15 cells were seeded overnight at a density of 8 × 10^5^ cells per well in a standard 6-well plate (BD Falcon). Media containing DMSO or 10 μM molecule 5 dissolved in DMSO (0.5% final concentration) were added to the cells and incubated for 72 h. Cellular DNA was extracted using the QIAamp DNA mini kit (Qiagen) and digested with plasmid-safe DNase (Lucigen)^28,77^. HBV cccDNA was quantified using premix Ex taq master mix (Takara) with taqman probe 5′-FAM-TTCAAGCCTCCAAGCTGTGCCTTGGGTGGC-TAMRA-3′ and real time primers FP: 5′-GTGCCTTCTCATCTGCCGG-3′ and RP: 5′-GAACTTTAGGCCCATATTAGTG-3′^77,78^. A standard curve was generated for absolute quantification^23^.

## Supplementary Information

Supplementary Information.
