# Supplementary material for: A novel piperazine derivative that targets hepatitis B surface antigen effectively inhibits tenofovir resistant hepatitis B virus
Source: Sci Rep. 2021 Jun 3;11:11723. doi: 10.1038/s41598-021-91196-1 (PMC8175705; doi:10.1038/s41598-021-91196-1)
Supplement: Supplementary file 1 — Supplementary Information. [file 41598_2021_91196_MOESM1_ESM.pdf]

Supplementary Information

A Novel Piperazine derivative that targets Hepatitis B Surface Antigen effectively inhibits Tenofovir Resistant Hepatitis B Virus

S. Kiruthika[1¶], Ruchika Bhat [2,3¶], Rozaleen Dash[4], Anurag S. Rathore[4], Perumal Vivekanandan[1*], B. Jayaram[1,2,3*]

[1]Kusuma School of Biological Sciences, Indian Institute of Technology Delhi, New Delhi, India

[2]Department of Chemistry, Indian Institute of Technology Delhi, New Delhi, India

[3]Supercomputing Facility for Bioinformatics & Computational Biology, Indian Institute of Technology Delhi, New Delhi, India

[4]Department of Chemical Engineering, Indian Institute of Technology Delhi, New Delhi, India

Corresponding authors

*Email: Perumal Vivekanandan, vperumal@bioschool.iitd.ac.in Phone: 91-11-26597532

*Email: B. Jayaram, bjayaram@chemistry.iitd.ac.in Phone: +91-11-26591505

¶These authors contributed equally to this work.

Table of Contents

Section	Title	Page no.
Figure S1.	2D structures of Tenofovir, Entecavir and ZINC20451377 (Molecule 5).	4
Figure S2.	Final predicted modeled structure of HBsAg.	5
Figure S3.	Cα backbone RMSD plot of all five complexes (HBsAg protein with each small molecule) shown for initial 10 ns of molecular dynamics simulations.	6
Figure S4.	Cell viability determined by MTT in presence of (A) Molecule 3 and (B) Molecule 5.	7
Figure S5.	Inhibition of secreted surface antigen (HBsAg) expression by Molecule 3, and 5 at 50μM concentration.	8
Figure S6	Cell viability in presence of molecule 5 determined by (A) cell counting kit-8 and (B) resazurin reduction assay.	9
Figure S7.	Relative Luciferase expression in Huh7 cells transfected with firefly luciferase reporter and renilla luciferase control plasmids in 10:1 ratio following 48 hours incubation with 10 μM of molecule 5.	10
Figure S8.	Surface plasmon resonance (SPR) analysis showing binding kinetics of Hepatitis B Surface Antigen (HBsAg) with ciclopirox.	11
Figure S9.	Dose-response curve for inhibition of wild type HBV encoded secreted HBV surface antigen (HBsAg) by lamivudine.	12
Figure S10.	(A) Inhibition of intracellular HBsAg expressed from 1.3×HBV construct and (B) secreted HBsAg expression from sub-genomic PreS2/S construct (C) intracellular HBsAg expressed from sub-genomic PreS2/S construct by Molecule 5 at 10μM concentration.	13
Figure S11.	Convergence plots of ligand RMSD (red) and Cα backbone atoms of protein-ligand complex (black) of the molecule 5 with HBsAg.	14
Figure S12	Hydrogen bond distance between protein and ligand atoms for Molecule 5 in complex with HBsAg.	15
Figure S13	Inhibition of hepatitis B virion secretion from wild type 1.3×HBV by 5μM lamivudine normalised to untreated control.	16
Figure S14	Inhibition of hepatitis B virion secretion from HepG2.2.15 (HBV stable expression cells) by 10μM molecule 5 normalised to DMSO control	17
Figure S15	HBV covalently closed circular DNA (ccc DNA) levels of HepG2.2.15 after treatment with 10μM molecule 5 normalised to DMSO control	18

Table S1.	List of top 150 screened ZINC small molecules with their binding affinity (kcal/mol) obtained using RASPD.	19
Table S2.	List of 30 ZINC small molecules which had best docking scores among the top 150 screened molecules. Predicted free binding energies (kcal/mol) for all the 30 molecules against HBsAg predicted using ParDOCK, AutoDock and SwissDock.	21
Table S3.	Structures of five proposed small molecules based on best binding energies obtained by ParDOCK docking software and their interaction patterns.	24
Table S4.	Values of kinetic rate constants and goodness of fit parameters obtained by SPR experiment.	26
Table S5.	IC50 values (μM) for Wild type (Wt) and lamivudine-resistant (rtM204I) mutant and tenofovir-resistant (CYEI) mutant HBV encoded secreted HBsAg.	27
Table S6.	ADMET properties of molecule 5 using SwissADME	28
Table S7.	Predicted binding free energies for Molecule 5 against HBsAg calculated using MMBAPPL and using AMBER (for average values during MD simulations).	29
Table S8.	Structural similarity based on Tanimoto coefficients between chemical compounds identified in published reports and molecule 5 identified in this study.	30
	References	31

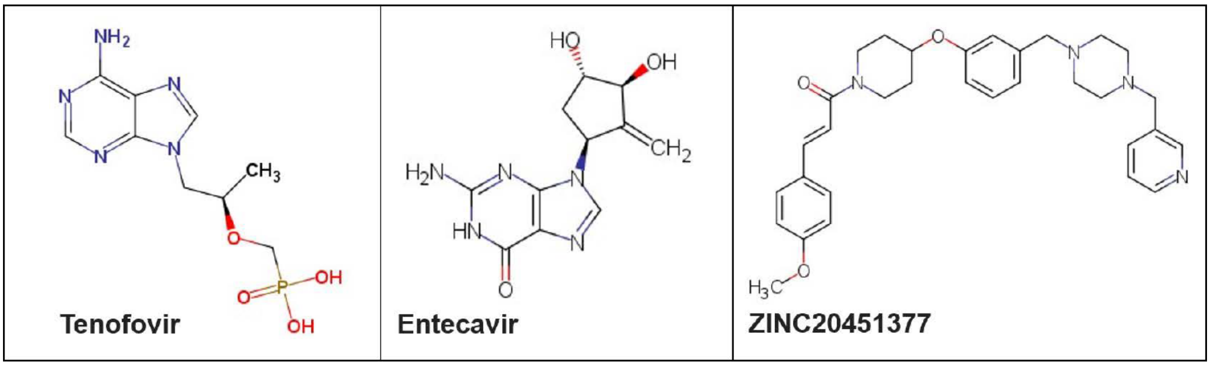

Figure S1. 2D structures of Tenofovir, Entecavir and ZINC20451377 (Molecule 5).

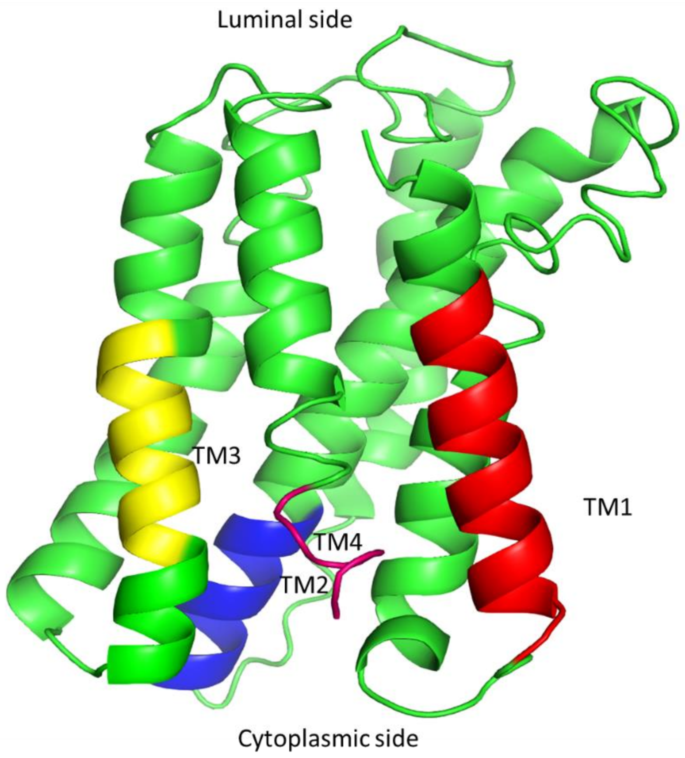

Figure S2. Final predicted modelled structure of HBsAg with luminal and cytoplasmic sides and membrane domains highlighted. Red (TM1), Blue (TM2), Yellow (TM3) and Pink (TM4). Models provided by Suffner *et al.* and Siegler *et al.* were used to find the domains in our model[1,2].

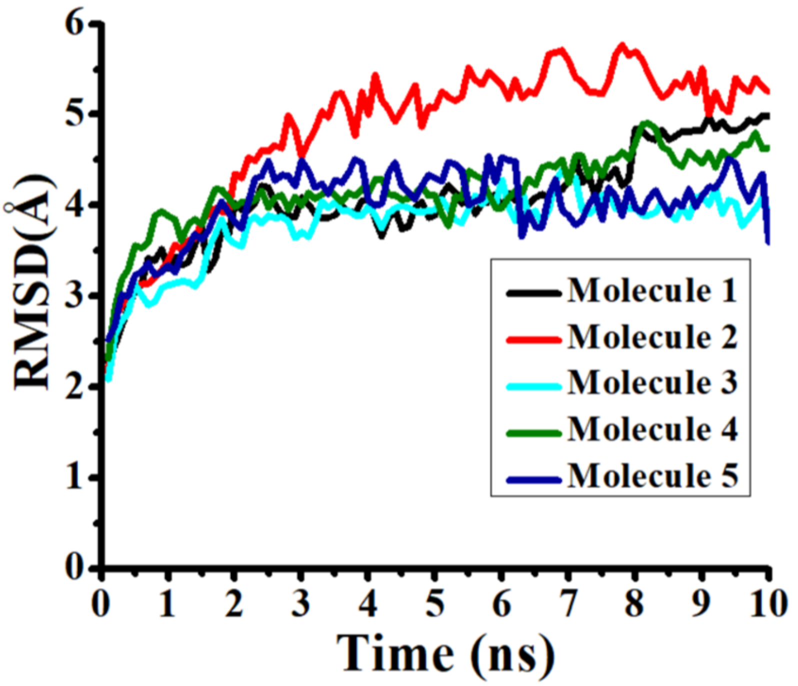

Figure S3. Cα backbone RMSD plot of all five complexes (HBsAg protein with each small molecule) shown for initial 10 ns of molecular dynamics simulations. As observed by the plot only molecules 3 and 5 showed overall stable backbone RMSDs whereas, molecules 1, 2, and 4 showed increase in RMSD after 8 ns.

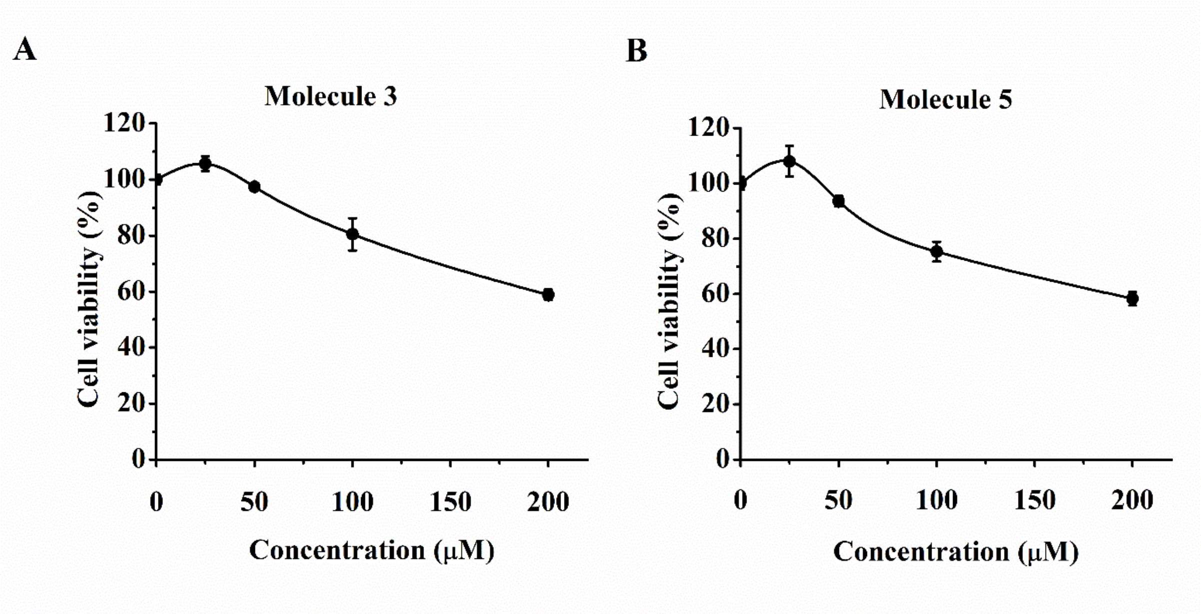

Figure S4. Cell viability determined by MTT in presence of (A) Molecule 3 and (B) Molecule 5. Huh7 cells were treated with increasing concentration of molecules dissolved in DMSO (0.5% final concentration) for 48 hours. MTT assay was performed in triplicates and data are represented as Mean ± SD.

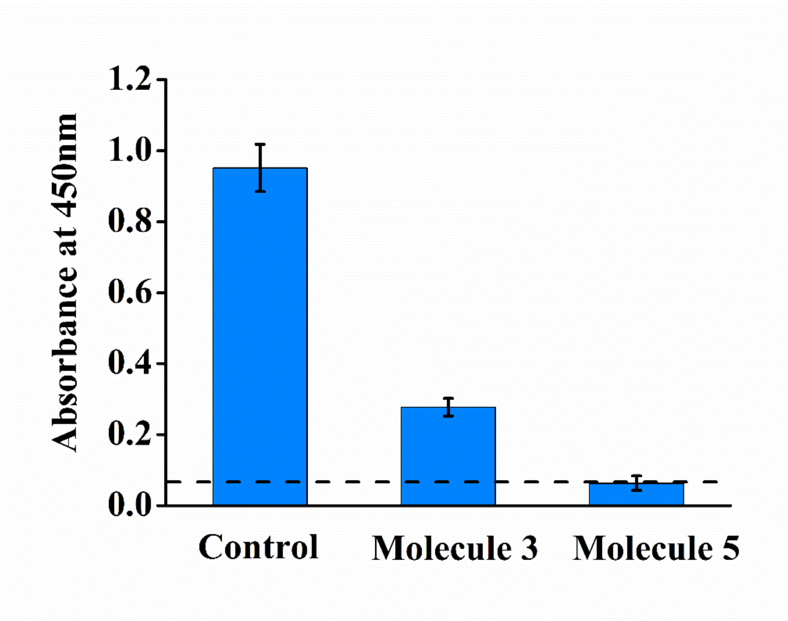

Figure S5. Inhibition of secreted surface antigen (HBsAg) expression by Molecule 3, and 5 at 50µM concentration. HBV surface antigen ELISA. Absorbance values obtained on testing culture supernatant are shown here. Cutoff value indicated by a dashed line was 0.0667

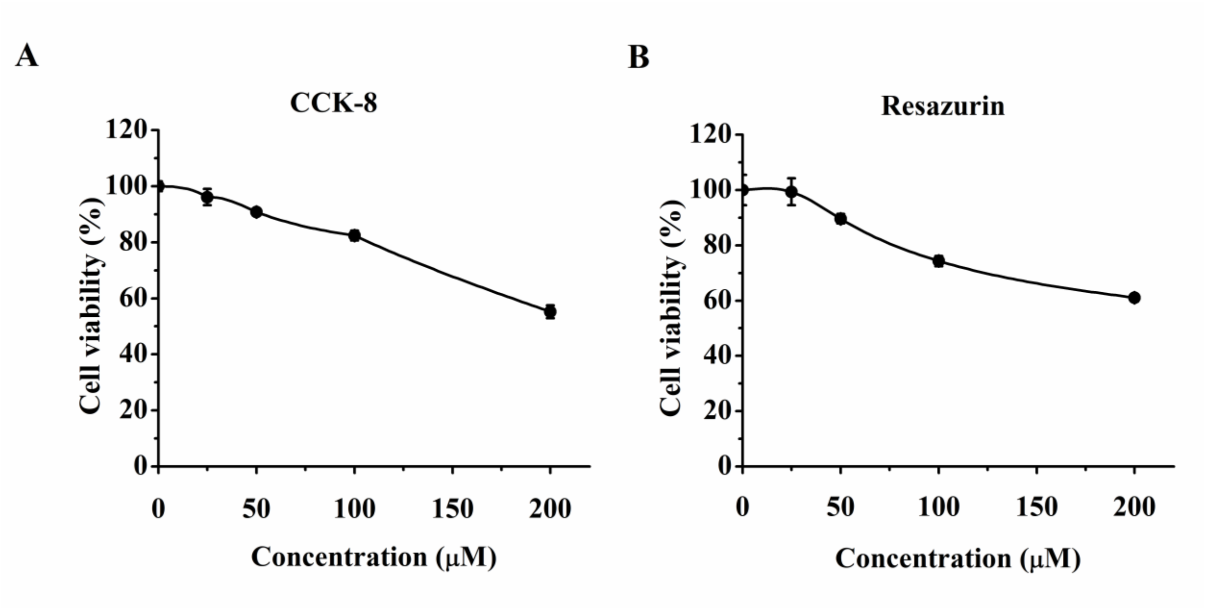

Figure S6. Cell viability in presence of molecule 5 determined by (A) cell counting kit-8 and (B) resazurin reduction assay. Huh7 cells were treated with increasing concentration of molecules dissolved in DMSO (0.5% final concentration) for 48 hours. Cell viability assay was performed in triplicates and data are represented as Mean ± SD.

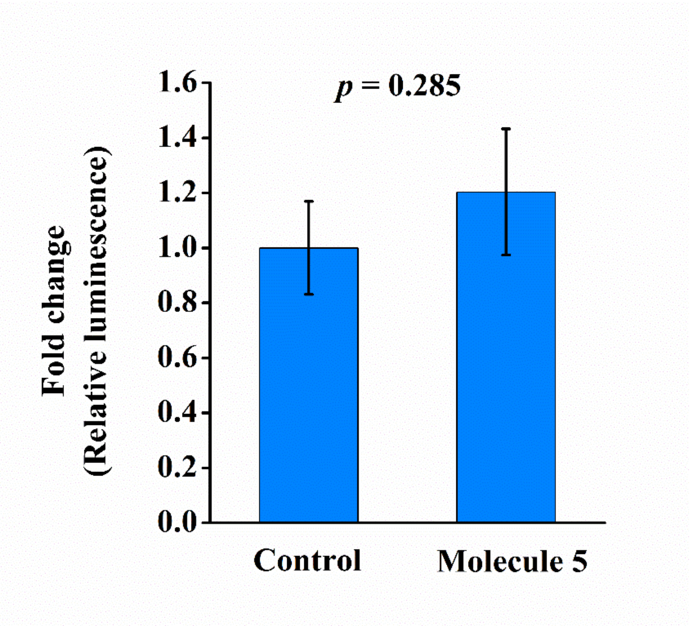

Figure S7. Relative Luciferase expression in Huh7 cells transfected with firefly luciferase reporter and renilla luciferase control plasmids in 10:1 ratio following 48 hours incubation with 10 μM of molecule 5. Luciferase assay was performed in triplicates and data are represented as Mean ± SD.

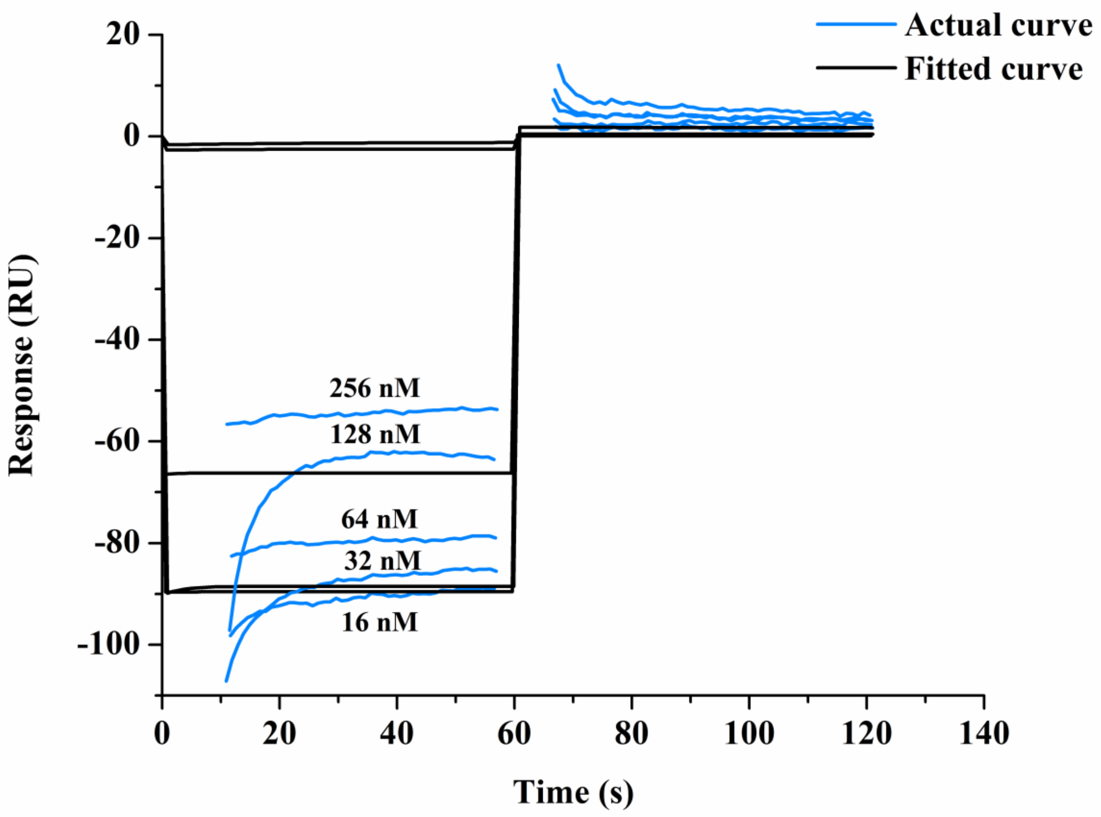

Figure S8. Surface plasmon resonance (SPR) analysis showing binding kinetics of Hepatitis B Surface Antigen (HBsAg) with ciclopirox. Kinetic analysis of HBsAg-ciclopirox binding was performed by injecting different known concentrations of ciclopirox (from 16 to 256 nM) over HBsAg immobilized carboxymethyl dextran-coated CM5 sensor chip (Amine-coupling chemistry). All measurements were performed at 25°C with a flow rate of 30 μL/min using HBS-EP buffer with association time 60s followed by 60s dissociation phase. Kinetic constants were calculated from the sensorgrams using the 1:1 fit model using BIA Evaluation 2.0.1 (Cytiva) software. The blue line indicates the actual curve and black line indicates the fitted curve of the sensorgram.

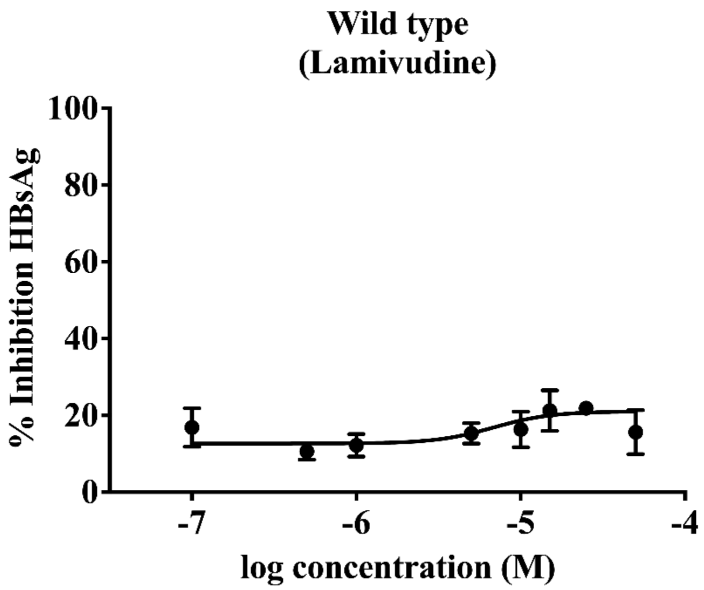

Figure S9. Dose-response curve for inhibition of wild type HBV encoded secreted HBV surface antigen (HBsAg) by lamivudine.

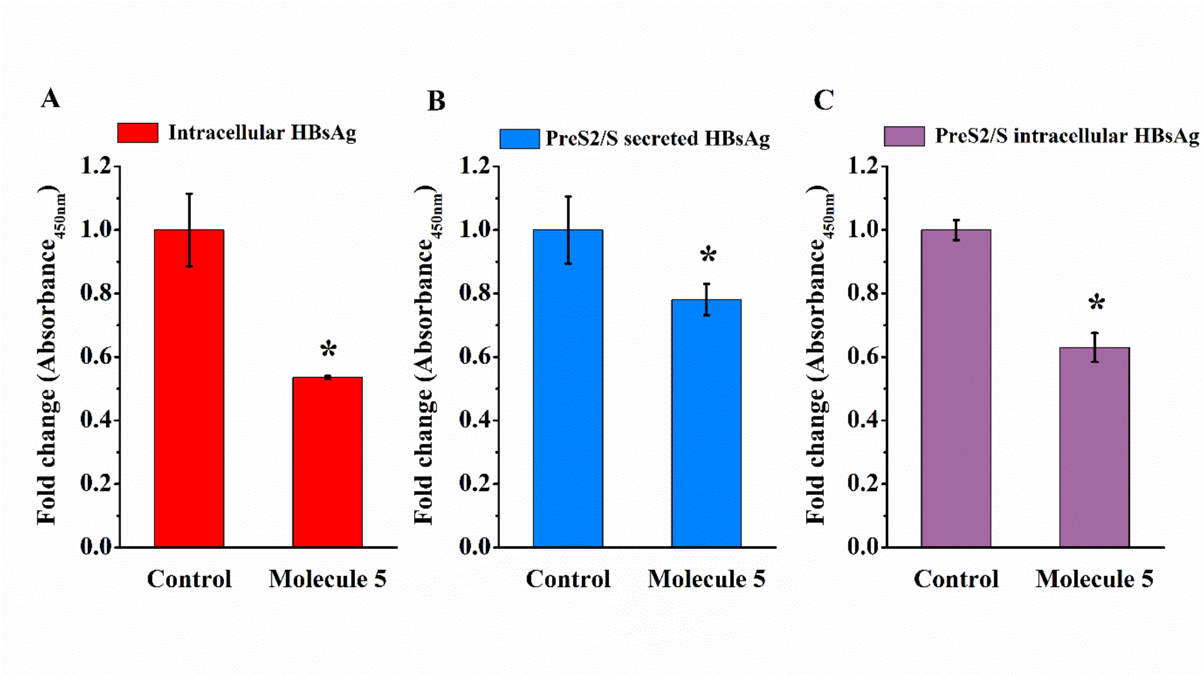

Figure S10. (A) Inhibition of intracellular HBsAg expressed from 1.3×HBV construct and (B) secreted HBsAg expressed from sub-genomic PreS2/S construct (C) intracellular HBsAg expressed from sub-genomic PreS2/S construct by Molecule 5 at 10µM concentration. Absorbance values obtained on testing cellular lysate/culture supernatant using HBV surface antigen ELISA normalized to control are shown here. Values significantly different from controls are indicated by a Student's t-test where, * p <0.05.

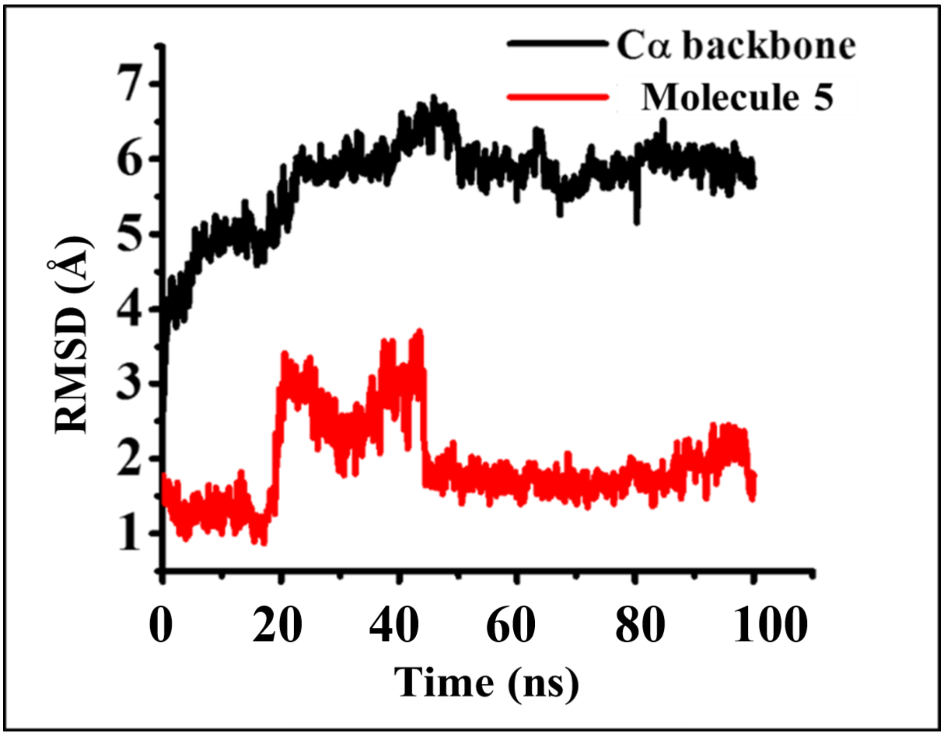

Figure S11. Convergence plots of ligand RMSD (red) and Cα backbone atoms of protein-ligand complex (black) of the molecule 5 with HBsAg. The plots are generated using Origin 4.0.

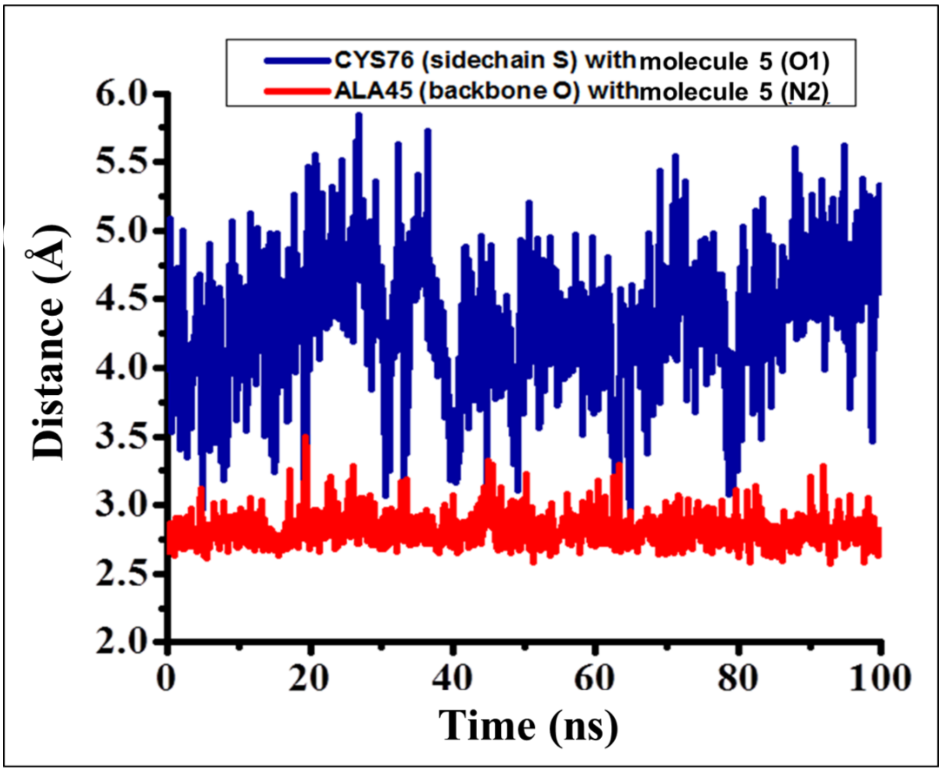

Figure S12. Hydrogen bond distance between protein and ligand atoms for Molecule 5 in complex with HBsAg.

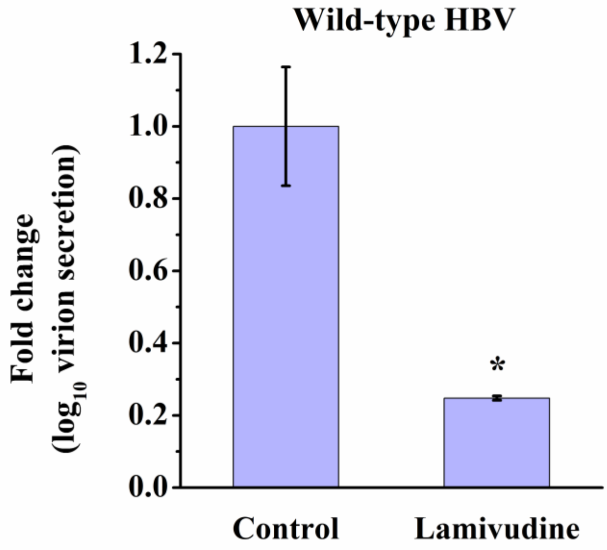

Figure S13. Inhibition of Secreted hepatitis B virion (log_{10} copies)/ml supernatant from wild type 1.3×HBV by 5µM lamivudine normalised to untreated control was estimated using real-time PCR following virion capture as described in the methods section. Values significantly different from controls are indicated by a Student's t-test where, * $p < 0.05$.

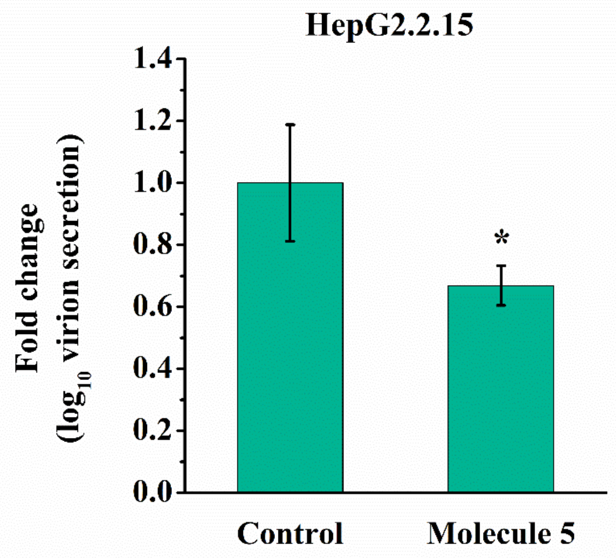

Figure S14 Inhibition of Secreted hepatitis B virion (log_{10} copies)/ml supernatant from HepG2.2.15 (HBV stable expression cells) by 10µM molecule 5 normalised to DMSO control (0.5% final concentration) was estimated using real-time PCR following virion capture as described in the methods section. Values significantly different from controls are indicated by a Student's t-test where, * $p < 0.05$.

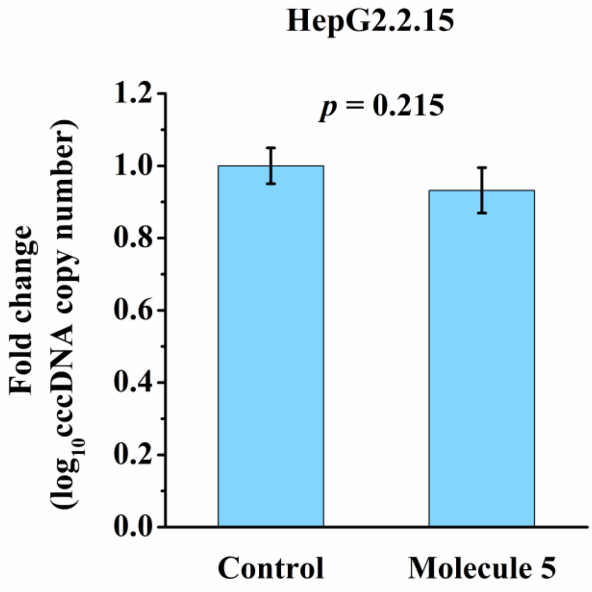

Figure S15. HBV covalently closed circular DNA (cccDNA) levels (log_{10} copies) of HepG2.2.15 after treatment with 10µM molecule 5 normalised to DMSO control (0.5% final concentration) was estimated using a Taqman probe based real-time PCR as described in the methods section.

Table S1. List of top 150 screened ZINC small molecules with their binding affinity (kcal/mol) obtained using RASPD.

Sr.No.	Binding Affinity	ZINC IDs	Sr.No.	Binding Affinity	ZINC IDs	Sr.No.	Binding Affinity	ZINC IDs
1	-14.5	ZINC16667348	51	-13.1	ZINC08442648	101	-12.8	ZINC09850497
2	-14.5	ZINC20601870	52	-13.1	ZINC09261062	102	-12.8	ZINC11785819
3	-14	ZINC02836173	53	-13.1	ZINC11840986	103	-12.8	ZINC11910201
4	-14	ZINC08892130	54	-13.1	ZINC11882026	104	-12.8	ZINC11997969
5	-14	ZINC12576410	55	-13.1	ZINC15823058	105	-12.8	ZINC12098176
6	-14	ZINC19797529	56	-13	ZINC01109335	106	-12.8	ZINC12151265
7	-14	ZINC19805326	57	-13	ZINC01216760	107	-12.8	ZINC12167142
8	-13.9	ZINC00793735	58	-13	ZINC02843658	108	-12.8	ZINC12191590
9	-13.9	ZINC08935093	59	-13	ZINC02983938	109	-12.8	ZINC12243297
10	-13.9	ZINC12050585	60	-13	ZINC02985067	110	-12.8	ZINC12245742
11	-13.8	ZINC03143011	61	-13	ZINC09226916	111	-12.8	ZINC12247497
12	-13.8	ZINC11913294	62	-13	ZINC09833600	112	-12.8	ZINC12572103
13	-13.7	ZINC01139950	63	-13	ZINC11980941	113	-12.8	ZINC12845408
14	-13.7	ZINC16193214	64	-13	ZINC12243261	114	-12.8	ZINC14882944
15	-13.7	ZINC19835705	65	-13	ZINC12243638	115	-12.8	ZINC14978739
16	-13.7	ZINC19857639	66	-13	ZINC12333572	116	-12.8	ZINC14986690
17	-13.6	ZINC02880085	67	-13	ZINC14952540	117	-12.8	ZINC14994985
18	-13.6	ZINC08680620	68	-13	ZINC19774479	118	-12.8	ZINC15000461
19	-13.6	ZINC11912462	69	-12.9	ZINC01067619	119	-12.8	ZINC15000762
20	-13.6	ZINC16248648	70	-12.9	ZINC01142376	120	-12.8	ZINC15008014
21	-13.6	ZINC19797618	71	-12.9	ZINC01807072	121	-12.8	ZINC16192643
22	-13.5	ZINC01799612	72	-12.9	ZINC02822264	122	-12.8	ZINC16993730
23	-13.5	ZINC02942316	73	-12.9	ZINC02895117	123	-12.8	ZINC19334116
24	-13.5	ZINC04020431	74	-12.9	ZINC02992414	124	-12.8	ZINC19741044
25	-13.5	ZINC12213012	75	-12.9	ZINC03656658	125	-12.8	ZINC20272398
26	-13.5	ZINC13322128	76	-12.9	ZINC06239462	126	-12.8	ZINC20451377

27	-13.5	ZINC14885566	77	-12.9	ZINC09970930	127	-12.8	ZINC22064288
28	-13.4	ZINC05221544	78	-12.9	ZINC11789550	128	-12.7	ZINC01092399
29	-13.4	ZINC12205684	79	-12.9	ZINC11791105	129	-12.7	ZINC02055756
30	-13.4	ZINC13081002	80	-12.9	ZINC11791473	130	-12.7	ZINC02063311
31	-13.4	ZINC13424926	81	-12.9	ZINC11881950	131	-12.7	ZINC04044463
32	-13.3	ZINC01158015	82	-12.9	ZINC11910416	132	-12.7	ZINC08905208
33	-13.3	ZINC01903996	83	-12.9	ZINC11974393	133	-12.7	ZINC09019193
34	-13.3	ZINC02744150	84	-12.9	ZINC12166642	134	-12.7	ZINC09123736
35	-13.3	ZINC09191993	85	-12.9	ZINC12219030	135	-12.7	ZINC09191244
36	-13.3	ZINC11784161	86	-12.9	ZINC12243664	136	-12.7	ZINC19809262
37	-13.3	ZINC11908856	87	-12.9	ZINC12246862	137	-12.7	ZINC09891916
38	-13.3	ZINC12492235	88	-12.9	ZINC14880667	138	-12.7	ZINC11787288
39	-13.3	ZINC16275934	89	-12.9	ZINC14992522	139	-12.7	ZINC11841585
40	-13.3	ZINC16480347	90	-12.9	ZINC14998924	140	-12.7	ZINC11881536
41	-13.2	ZINC02109070	91	-12.9	ZINC15005335	141	-12.7	ZINC11909120
42	-13.2	ZINC02760064	92	-12.9	ZINC15005994	142	-12.7	ZINC11913115
43	-13.2	ZINC03877717	93	-12.9	ZINC19758600	143	-12.7	ZINC12035725
44	-13.2	ZINC12243318	94	-12.9	ZINC19811543	144	-12.7	ZINC12135439
45	-13.2	ZINC12419770	95	-12.9	ZINC19840440	145	-12.7	ZINC12151796
46	-13.2	ZINC12727037	96	-12.8	ZINC00653293	146	-12.7	ZINC12210406
47	-13.2	ZINC14980650	97	-12.8	ZINC01161284	147	-12.7	ZINC14533264
48	-13.1	ZINC01234079	98	-12.8	ZINC02504256	148	-12.7	ZINC14743064
49	-13.1	ZINC01794178	99	-12.8	ZINC03877668	149	-12.7	ZINC14750468
50	-13.1	ZINC02851420	100	-12.8	ZINC08872344	150	-12.7	ZINC14883122

Table S2. List of 30 ZINC small molecules which had best docking scores among the top 150 screened molecules. Predicted free binding energies (kcal/mol) for all the 30 molecules against HBsAg predicted using ParDOCK, AutoDock and SwissDock.

Sr. No.	ZINC ID	IUPAC Names	ParDOCK	SwissDock	AutoDock
1	ZINC00653293	3,5-Pyridinediylbis{[4-(4-methoxyphenyl)-1-piperazinyl]methanone}	-11.5	-8.1	-7.1
2	ZINC11787288	(3S)-N-(1,3-Benzodioxol-5-ylmethyl)-1-(4-{2-[(4-ethylphenyl)amino]-2-oxoethoxy}phenyl)-5-oxo-3-pyrrolidinecarboxamide	-11.4	-9.2	-7.7
3	ZINC20451377	(2E)-3-(4-Methoxyphenyl)-1-[4-(3-{[4-(3-pyridinylmethyl)-1-piperazinyl]methyl}phenoxy)-1-piperidinyl]-2-propen-1-one	-11.1	-8.1	-8.3
4	ZINC19809262	N-(4-Ethoxyphenyl)-2-{1-(4-methoxyphenyl)-3-[2-(4-morpholinyl)ethyl]-5-oxo-2-thioxo-4-imidazolidinyl}acetamide	-10.8	-7.6	-6.7
5	ZINC19805326	N-(4-Butylphenyl)-N'-{2-[4-(2-naphthylsulfonyl)-1-piperazinyl]ethyl}ethanediamide	-10.9	-8.9	-7.0
6	ZINC11910201	4-(4-{[4-(4-Methylphenyl)-1-piperazinyl]carbonyl}-1-piperidinyl)-2-(3-pyridinylmethyl)-1H-isoindole-1,3(2H)-dione	-10.8	-8.8	-7.7
7	ZINC03877668	(5Z)-3-Ethyl-5-[(2E)-2-(3-ethyl-5-methoxy-1,3-benzothiazol-2(3H)-ylidene)ethylidene]-2-(3-methyl-5-oxo-1-phenyl-1,5-dihydro-4H-pyrazol-4-ylidene)-1,3-thiazolidin-4-one	-10.1	-8.4	-7.4
8	ZINC11913294	(4-Ethyl-1-piperazinyl)[4-(4-{[2-hydroxy-3-(3-methoxyphenoxy)propyl]amino}-1-piperidinyl)phenyl]methanone	-10.1	-9.0	-6.9
9	ZINC01794178	2-({5-[(1,3-Benzothiazol-2-ylsulfanyl)methyl]-4-methyl-4H-1,2,4-triazol-3-yl}sulfanyl)-N-[2-(3,4-dimethoxyphenyl)ethyl]acetamide	-9.8	-8.5	-6.4
10	ZINC12050585	N-[3-(4-{[4-(2-Pyrimidinyl)-1-piperazinyl]methyl}phenoxy)propyl]-4-biphenylcarboxamide	-9.1	-8.9	-6.7
11	ZINC04020431	3,5-Bis[4-(4-pentylcyclohexyl)phenyl]-1,2,4-oxadiazole	-8.8	-8.1	-6.2
12	ZINC16193214	(5Z)-3-(4-Fluorophenyl)-5-{4-[3-(3-methoxyphenoxy)propoxy]benzylidene}-2-thioxo-1,3-thiazolidin-4-one	-8.7	-8.6	-6.5
13	ZINC01109335	4-(Benzyloxy)-N-{4-[4-(2-thienylcarbonyl)-1-piperazinyl]phenyl}benzamide	-8.6	-7.8	-6.2

14	ZINC01139950	(3S)-N-(1,3-Benzodioxol-5-ylmethyl)-1-(4-{2-[(4-ethylphenyl)amino]-2-oxoethoxy}phenyl)-5-oxo-3-pyrrolidinecarboxamide	-8.2	-9.0	-7.0
15	ZINC02836173	2-(4-Methoxyphenyl)-N-{[4-methyl-5-({2-[(5-methyl-4-phenyl-1,3-thiazol-2-yl)amino]-2-oxoethyl}sulfanyl)-4H-1,2,4-triazol-3-yl]methyl}acetamide	-8.2	-8.1	-6.5
16	ZINC01092399	(5E)-5-({2,5-Dimethyl-1-[4-(4-morpholinyl)phenyl]-1H-pyrrol-3-yl}methylene)-1-(4-methoxyphenyl)-2-thioxodihydro-4,6(1H,5H)-pyrimidinedione	-8.1	-8.3	-6.3
17	ZINC05221544	N-{(1S)-1-[(2R)-2,3-Dihydro-1,4-benzodioxin-2-yl]ethyl}-N'-{(1S)-1-[(2S)-2,3-dihydro-1,4-benzodioxin-2-yl]ethyl}decanediamide	-8.1	-8.5	-6.5
18	ZINC16667348	N'1,N'9-Bis{(E)-[4-(diethylamino)phenyl]methylene}nonanedihydrazide	-8.1	-9.0	-7.2
19	ZINC03143011	(2E,2'E)-N,N'-(2,5-Pyrimidinediyldi-4,1-phenylene)bis(3-phenylacrylamide)	-8.0	-8.0	-6.1
20	ZINC08680620	(4-Benzyl-1-piperazinyl)[5-(2-thienyl)-7-(trifluoromethyl)pyrazolo[1,5-a]pyrimidin-3-yl]methanone	-8.0	-8.1	-6.7
21	ZINC01067619	2-{[6-(1,3-Dioxo-1,3-dihydro-2H-isoindol-2-yl)-1,3-benzothiazol-2-yl]sulfanyl}-N-(4-ethoxyphenyl)acetamide	-7.9	-8.5	-6.1
22	ZINC08892130	2-(5-{4-[(2-Fluorobenzyl)oxy]phenyl}-2H-tetrazol-2-yl)-N-[3-(hexyloxy)phenyl]acetamide	-7.9	-9.1	-6.7
23	ZINC19797618	N-(4-Butylphenyl)-N'-(2-{4-[(4-methylphenyl)sulfonyl]-1-piperazinyl}ethyl)ethanediamide	-7.9	-8.2	-7.1
24	ZINC02880085	4-Bromo-N-{2-[5-({2-[(4-ethoxyphenyl)amino]-2-oxoethyl}sulfanyl)-4-methyl-4H-1,2,4-triazol-3-yl]ethyl}benzamide	-7.7	-8.1	-6.8
25	ZINC19797529	(3S)-1-[4-(Hexyloxy)phenyl]-3-{4-[(2E)-3-phenyl-2-propen-1-yl]-1-piperazinyl}-2,5-pyrrolidinedione	-7.6	-8.6	-6.3
26	ZINC00793735	(4-Benzyl-1-piperazinyl)[5-(2-thienyl)-7-(trifluoromethyl)[1,2,4]triazolo[1,5-a]pyrimidin-2-yl]methanone	-7.3	-7.8	-6.1
27	ZINC20601870	5-(2-Furyl)-N-{1-[2-(1-piperidinyl)ethyl]-1H-benzimidazol-2-yl}-7-(trifluoromethyl)pyrazolo[1,5-a]pyrimidine-3-carboxamide	-7.3	-7.5	-6.3

28	ZINC16248648	2-(Methylsulfanyl)ethyl {4-[(E)-{[4-(4-morpholinylmethyl)benzoyl]hydrazono}methyl]phenoxy}acetate	-7.2	-7.7	-6.3
29	ZINC08935093	Bis[4-(hexyloxy)phenyl] spiro[3.3]heptane-2,6-dicarboxylate	-7.1	-8.7	-5.9
30	ZINC12576410	2,2'-{3,6-Acridinediylbis[nitrilo(E)methylylidene]}bis(4-nitrophenol)	-6.9	-7.7	-6.6

Table S3. Structures of five proposed small molecules based on best binding energies obtained by ParDOCK docking software and their interaction patterns.

**Name**	**Chemical Structure**	**Interactions with HBsAg**
**Molecule 1 (ZINC00653293)**	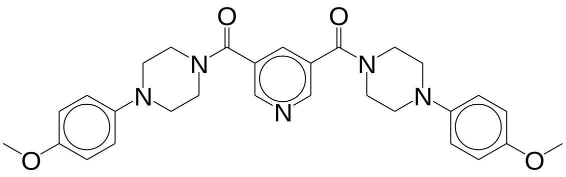	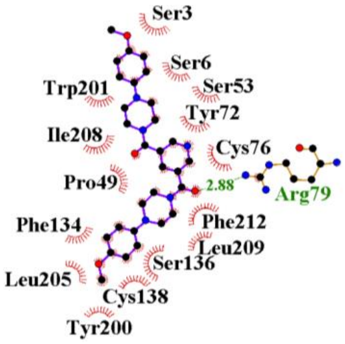
**Molecule 2 (ZINC11787288)**	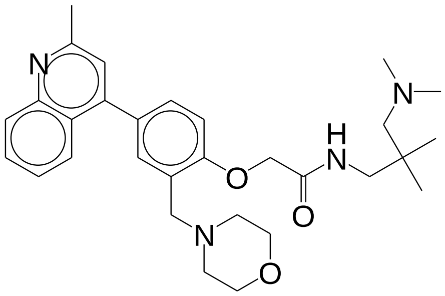	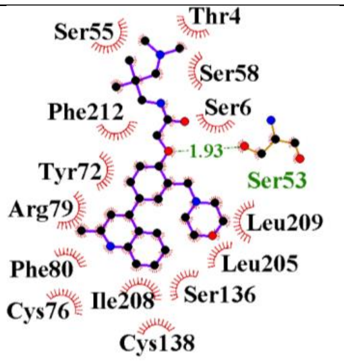
**Molecule 3 (ZINC19809262)**	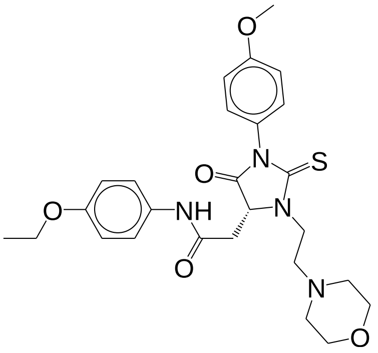	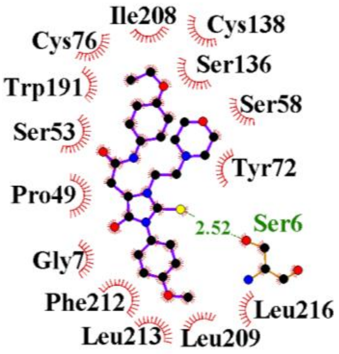

<table>
<tr>
<td>Molecule 4
(ZINC19805326)</td>
<td>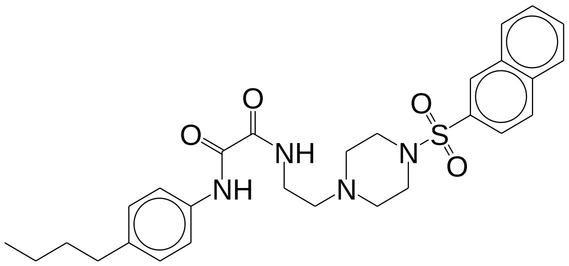</td>
<td>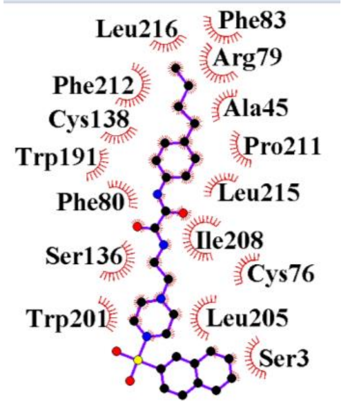
</td>
</tr>
<tr>
<td>Molecule 5
(ZINC20451377)</td>
<td>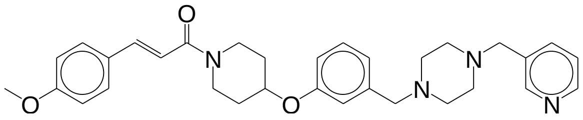</td>
<td>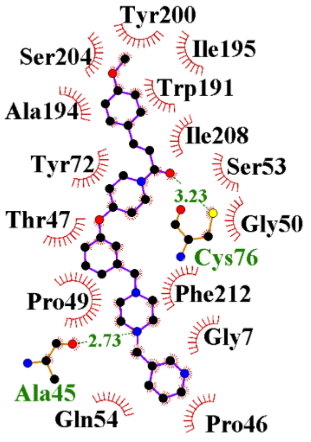
</td>
</tr>
</table>

Table S4. Values of kinetic rate constants and goodness of fit parameters obtained by SPR experiment.

	**ka (1/Ms)**	**kd (1/s)**	**KD (M)**	**Chi² (RU²)**	**U-value**
**Ciclopirox (Negative control)**	2.42×10^{6}	6.57×10^{-4}	2.72×10^{-10}	531	95
**Molecule 5**	6.21×10^{5}	0.04057	6.53×10^{-8}	0.192	3

Table S5. IC_{50} values (μM) for Wild type (Wt) and lamivudine-resistant (rtM204I) mutant and tenofovir-resistant (CYEI) mutant HBV encoded secreted HBsAg.

**HBV**	**Wt**	**rtM204I**	**CYEI**
**IC50 for molecule 5 (μM)**	20.84	5.561	11.39

Table S6. ADMET properties of molecule 5 using SwissADME [3].

Molecule	GI[#] absorption [4]	BBB[#] permeability [4]	Number of drug likeliness rules followed*
Molecule 5	High	Yes	Lipinski : 1 Violation; MW > 500<br><br>Ghose : 3 Violations; MW > 480, MR > 130, No.of atoms > 70<br><br>Veber : Follows (0 Violations)<br>Egan : Follows (0 Violations)<br><br>Muegge : Follows (0 Violations)

*Lipinski Rule[5]: MW ≤ 500; MLOGP ≤ 4.51; No. of acceptors ≤ 10; No. of donors ≤ 5.

*Ghose Rule[6]: 160 ≤ MW ≤ 480; -0.4 ≤ WLOGP ≤ 5.6; 40 ≤ MR ≤ 130; 20 ≤ No. of atoms ≤ 70.

*Veber Rule[7]: Rotatable bonds ≤ 10; TPSA ≤ 140.

*Egan Rule: WLOGP ≤ 5.88; TPSA ≤ 131.6.

*Muegge Rule[8]: 200 ≤ MW ≤ 600; -2 XLOGP ≤ 5; TPSA ≤ 150; No. of rings ≤ 7; No. of C >4; No. of heteroatoms > 1; No. of rotatable bonds ≤ 15; No. of acceptors ≤ 10; No. of donors ≤ 5.

[#]GI: Gastrointestinal absorption; BBB: Blood brain barrier permeation.

Table S7. Predicted binding free energies for Molecule 5 against HBsAg calculated using MMBAPPL and using AMBER (for average values during MD simulations).

**Molecule**	**ZINC ID**	**MMBAPPL Score (kcal/mol)**	**MMGBSA (kcal/mol)**	**MMPBSA (kcal/mol)**
**Molecule 5**	**ZINC20451377**	-8.19	-50.01	-16.99

Table S8. Structural similarity based on Tanimoto coefficients between chemical compounds identified in published reports and molecule 5 identified in this study.

Molecule	Nicotinamide	BM601	NJK14047	HBF-0259
**Molecule 5**	0.13	0.09	0.11	0.21

References

1. Suffner, S. *et al.* Domains of the Hepatitis B Virus Small Surface Protein S Mediating Oligomerization. *J. Virol.* **92**, 1–15 (2018).

2. Siegler, V. D. & Bruss, V. Role of Transmembrane Domains of Hepatitis B Virus Small Surface Proteins in Subviral-Particle Biogenesis. *J. Virol.* **87**, 1491–1496 (2013).

3. Daina, A., Michielin, O. & Zoete, V. SwissADME: a free web tool to evaluate pharmacokinetics, drug-likeness and medicinal chemistry friendliness of small molecules. *Sci. Rep.* **7**, 42717 (2017).

4. Daina, A. & Zoete, V. A BOILED-Egg To Predict Gastrointestinal Absorption and Brain Penetration of Small Molecules. *ChemMedChem* **11**, 1117–1121 (2016).

5. Lipinski, C. A., Lombardo, F., Dominy, B. W. & Feeney, P. J. Experimental and computational approaches to estimate solubility and permeability in drug discovery and development settings1PII of original article: S0169-409X(96)00423-1. The article was originally published in Advanced Drug Delivery Reviews 23 (1997) 3. *Adv. Drug Deliv. Rev.* **46**, 3–26 (2001).

6. Ghose, A. K., Viswanadhan, V. N. & Wendoloski, J. J. A Knowledge-Based Approach in Designing Combinatorial or Medicinal Chemistry Libraries for Drug Discovery. 1. A Qualitative and Quantitative Characterization of Known Drug Databases. *J. Comb. Chem.* **1**, 55–68 (1999).

7. Veber, D. F. *et al.* Molecular Properties That Influence the Oral Bioavailability of Drug Candidates. *J. Med. Chem.* **45**, 2615–2623 (2002).

8. Muegge, I., Heald, S. L. & Brittelli, D. Simple Selection Criteria for Drug-like Chemical Matter. *J. Med. Chem.* **44**, 1841–1846 (2001).
